# Oxygen-Pressure Protocol Breaking Cycle Limit of Continuously Reversible Lithium-Oxygen Batteries

**DOI:** 10.1007/s40820-025-01990-z

**Published:** 2026-01-05

**Authors:** Xinhang Cui, Fenglong Xiao, Guoliang Zhang, Zhangliu Tian, Qingshan Bao, Yanlu Li, Deliang Cui, Qilong Wang, Feng Dang, Wei Chen, Haohai Yu, Huaijin Zhang, Gang Lian

**Affiliations:** 1 State Key Laboratory of Crystal Materials, Shandong University, Jinan, 250100 People’s Republic of China; 2 School of Materials Science and Engineering, Shandong University, Jinan, 250061 People’s Republic of China; 3 Department of Physics, National University of Singapore, 2 Science Drive 3, Queenstown, 117543 Singapore; 4 Key Laboratory for Special Functional Aggregated Materials of Education Ministry, School of Chemistry and Chemical Engineering, Shandong University, Jinan, 250100 People’s Republic of China

**Keywords:** Li-O_2_ batteries, O_2_ pressure, Cycle life, Li anode protection, Rate performance

## Abstract

Lithium-oxygen (Li-O_2_) battery is favored among “beyond lithium-ion” technologies for sustainability because of its exceptional energy density. Major impediments are the poor cycle stability and grievous capacity degradation at high current densities. We address these issues by a “killing two birds with one stone” O_2_-pressure protocol. It first resolves efficient O_2_ mass transport at high rates.æ The accelerated reaction kinetics optimizes the composition and growth pathway of discharge products. This protocol secondly achieves protection of Li anodes via densifying corrosion layers on them. Consequently, the battery delivers both ultrahigh discharge capacity (> 9,000 mAh g^−1^) at 3,000 mA g^−1^ and excellent cycling stability. Under a dual-strategy effect of high-pressure O_2_ and artificial protection layers, the battery actualizes over 11-fold increase in cycle life of 5,170 h (2,585 cycles). The strategy opens avenues for advancing Li-O_2_ batteries towards practical application and confers the extension to other gas-based batteries.

**Supplementary Information:**

The online version contains supplementary material available at 10.1007/s40820-025-01990-z.

## Highlights

- An O_2_− pressure protocol was proposed to strengthen mass transport, accelerate the reaction kinetics and optimize growth pathways of discharge products, which achieves ultrahigh discharge capacity at 3,000 mA g^−1^ (>9,000 mAh g^−1^).
- This general pressure effect can protect Li anodes via densifying corrosion layers on them simultaneously.
- The breakthrough of continuously operated ultralong-life lithium-oxygen batteries was actualized over a record-high lifetime of ~5,170 h (2,585 cycles) at 500 mA g^−1^ under constant operation.

**Supplementary Information:**

The online version contains supplementary material available at 10.1007/s40820-025-01990-z.

## Graphical abstract

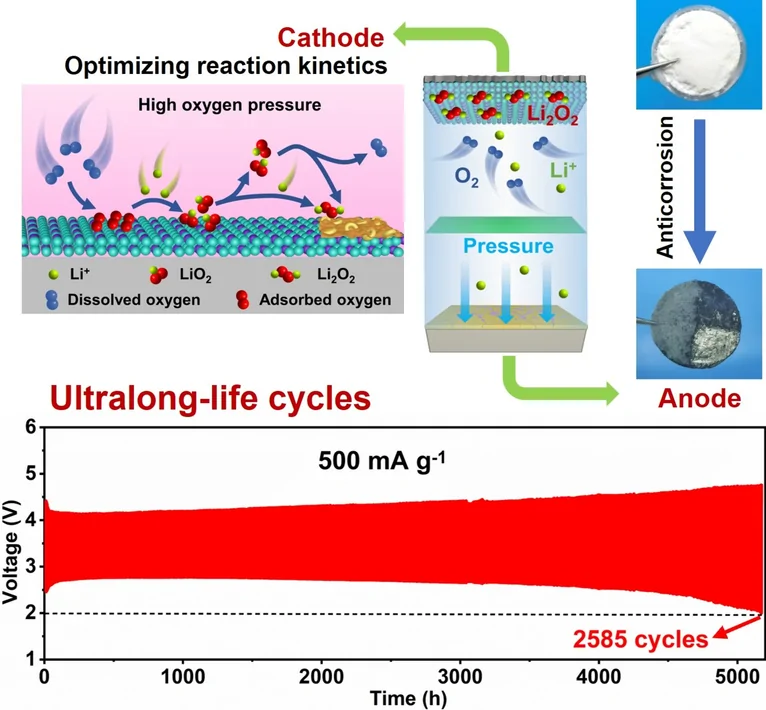

## Introduction

In the quest for sustainable energy solutions, redox chemistries based on oxygen (O_2_) are gaining prominence because they diminish reliance on limited transition metal elements and promise high energy density (≈3,500 Wh kg^−1^) when paired with lithium (Li) anode [1–6]. Typical Li-O_2_ battery (LOB) chemistry involves reversible formation and decomposition of Li_2_O_2_ at the cathode, wherein sluggish reaction kinetics and severe corrosion of Li anode result in inferior cycle life and rate capability [7]. Despite the efficient cathode materials [8–12], the corresponding conversion generally exhibits limited capacities and poor cycle stability at high current densities, which are mainly caused by the “altitude sickness” of cathode and poor charge transfer between cathode and insulating discharge products. Incomplete conversion of Li_2_O_2_ accelerates continuous accumulation of it and passivates catalysis sites, further diminishing the limited capacity and impeding cycling stability. Because O_2_ solubility in organic electrolytes is far lower than the concentration of Li ions [13], O_2_ supply is the rate limiting factor of forming discharge products (2Li^+^  + O_2_ + 2e^−^ → Li_2_O_2_). In addition, large charge potentials are generally required to oxidize the insulating Li_2_O_2_ deposits. Therefore, the formation of Li-vacancy-type Li_2_O_2_ (Li_2-x_O_2_) with high conductivity and poor crystallinity are expected during circular operation of LOBs. O_2_-rich environment is effective to address this concern. Because O_2_ solubility in electrolyte generally increases with external O_2_ pressure, high O_2_ pressure should be an efficient strategy to intrinsically accelerate reaction kinetics especially at high current densities, extend the cycle life and enhance capacity retention of LOBs.

Li anode corrosion is another severe problem to degrade the cycle life of LOBs. The formation of fluffy corrosion layer can’t prevent the shuttle effect of corrosion sources and rapid corrosion of unprotected Li anode. Much work has been conducted to protect the Li metal [14–24], mainly including inorganic layers, organic layers, and inorganic–organic hybrid layers. For inorganic layers, they generally present high mechanical strength and Li^+^ conductivity but relatively poor toughness. During the repeated dissolution and deposition of Li^+^, they easily crack due to volume change of anodes. It results in the loss of their protective effect. In contrast, organic protection layers generally have good flexibility, but they present poor Li^+^ conductivity and rigidity. The effect of effective Li^+^ transport and inhibiting the growth of Li dendrites is inferior compared to inorganic layers. Combining of their respective advantages, inorganic–organic hybrid layers are then designed to achieve the complementary advantages of them, resulting in more superior protective effect. Despite the development in mitigating Li corrosion to some extent, these multistep and time-/cost-consuming technical processes are still far from anticipated expansion of long-term battery operation. Accordingly, high-efficient protection of Li anodes is still a tough challenge. Undoubtedly, high pressure can densify the loose structure of lithium hydroxide (LiOH, main lithium corrosion product) [25]. The shuttle effect of harmful substances can be suppressed to fresh Li metal surface. The environment-friendly external O_2_-pressure-tuned approach should be a facile and general self-passivation strategy to realize corrosion mitigation of unprotected Li anode. Evidently, the dual-purpose O_2_-pressure strategy is a revolutionary approach towards high-rate and long-life LOBs. In spite of the effect of O_2_ pressure for the LOBs mentioned before [26–29], there is a lack of systematic research for significant enhancement of electrochemical performance especially for cathodes and anodes with dual positive purpose.

In this study, we demonstrate a novel prototype of O_2_-compensation LOBs that enable ultralong cycle life and large capacity retention especially at high current densities. The O_2_-pressure strategy achieves “killing two birds with one stone”. The first purpose is to satisfy the urgent need for abundant O_2_ components at high rates to accelerate reaction kinetics and optimize reaction pathway. Results present a large discharge capacity over 9,000 mAh g^−1^ at 3,000 mA g^−1^. The second purpose is to densify the LiOH corrosion layer to mitigate corrosion of Li anode. Under the dual-strategy effect of high O_2_ pressure and an artificial protection layer of Li anode, the battery actualizes an ultralong cycle life of 5,170 h (up to 2,585 cycles at 500 mA g^−1^). This concept offers a general optimization strategy of rate capacity and cycle life in metal-air battery fields.

## Exprimental Section

### Materials

Ruthenium (III) chloride hydrate (RuCl_3_·xH_2_O), Cobalt (II) chloride hexahydrate (CoCl_2_·6H_2_O), N-Methyl-2-pyrrolidone (NMP) and anhydrous ethanol (C_2_H_5_OH) were purchased from Aladdin Corporation. Glucose (C_6_H_12_O_6_) and Urea (CO(NH_2_)_2_) were purchased from Sinopharm Chemical Reagent Co., Ltd. Anhydrous tetraethylene glycol dimethyl ether (TEGDME) and lithium bis(trifluoromethane) sulfonamide (LiTFSI) were purchased from Suzhou Dodochem, China. Glass microfiber filters (GF/B, Whatman), Polyvinylidene fluoride (PVDF, Arkema) and Ketjen Black (KB, Lion Corporation) were purchased from other agents in China.

### Synthesis of Catalysts

Nitrogen-doped carbon-supported Co_3_Ru nanodots (NC/Co_3_Ru-NDs) were prepared by a modified method in our group [30]. Typically, 0.2 mmol ruthenium chloride (RuCl_3_), 0.2 mmol cobalt chloride (CoCl_2_·6H_2_O), 33.3 mmol glucose (C_6_H_12_O_6_) and 33.3 mmol urea (CO(NH_2_)_2_) were added in 10 ml deionized water to form a homogeneous solution. A hydrothermal reaction was proceeded at 150 °C for 10 h. After that, the precursor was obtained by washing the as-prepared powders with anhydrous ethanol and deionized water, and drying them at 80 °C for 24 h. The precursor was then annealed at 900 °C under Ar for 2 h. Finally, the NC/Co_3_Ru-NDs sample was obtained.

### Li Anodes Protection

The Li anode protection strategies referred to our previous work [30]. The detailed content is available in Supporting Information.

### Electrochemical Performance Measurements

#### Preparation of KB Cathodes

KB was mixed with PVDF in NMP with mass ratio of 8:2. The mixture is then dispersed in NMP and continuous stirring was applied for 12 h for the well-dispersed slurry. Then the prepared slurry was coated on carbon paper (TORAY, TGP-H-060, hydrophobic). The as-prepared cathode was heat-dried in vacuum at 110 °C for 12 h.

#### Preparation of Catalyst Cathodes

The cathode slurry was prepared by mixing catalyst (40 wt%), KB (40 wt%) and PVDF (20 wt%) into NMP. The slurry was uniformly coated on carbon paper to make the cathode and then heat-dried in vacuum at 110 °C for 12 h.

#### Battery Assembly

The Li-O_2_ batteries consist of an oxygen cathode, a fresh Li foil anode or protective Li anode, and a glass fiber separator dipped in a 1.0 M LiTFSI/TEGDME electrolyte. The batteries were assembled in a glove box filled with argon (H_2_O < 0.01 ppm, O_2_ < 0.01 ppm). To guarantee the constant high-pressure environment, all tests were performed in well-sealed specially designed chambers.

#### Electrochemical Testing

The LAND multi-channel battery tester (Wuhan Land Electronic Co., Ltd) operated the galvanostatic discharge/charge tests of the Li-O_2_ batteries. The current density and specific capacity were normalized by the calculated mass of catalyst. Cyclic voltammetry (CV) scanning was carried out on an electrochemical workstation (Chenhua, Shanghai, CHI760E) with a voltage range of 2.0–4.5 V. Electrochemical impedance spectroscopy (EIS) was also performed on this electrochemical workstation.

### Material Characterizations

Morphology images were acquired from field-emission scanning electron microscope (G300, Carl Zeiss) and white light interferometer (ZeGage Pro HR, ZYGO). X-ray diffractometer (SmartLab 9 Kw, Rigaku) was employed for crystal phase analysis. The surface element composition and combination states of catalysts were studied by X-Ray Photoelectron Spectroscopy (AXIS Supra, Kratos). Contact angles were collected by contact angle goniometer (JC2000D, Powereach). Raman spectra were recorded by Confocal Raman Microscope (DXR, Thermo − Fisher Scientific) with an excitation line of 633 nm. Fourier transformation infrared absorption spectra of the samples were recorded by using a Nicolet NEXUS 670 Fourier transformation infrared spectrometer, with a wavenumber resolution of 4 cm^−1^ (4000–650 cm^−1^).

### Solubility of Oxygen in Electrolyte Measurement and Computational Methods

The measurement of solubility of oxygen in electrolyte at different pressure and the related calculations are available in Supporting Information.

## Results and Discussion

### Design of O_2_-Compensation LOBs

**Fig. 1:**
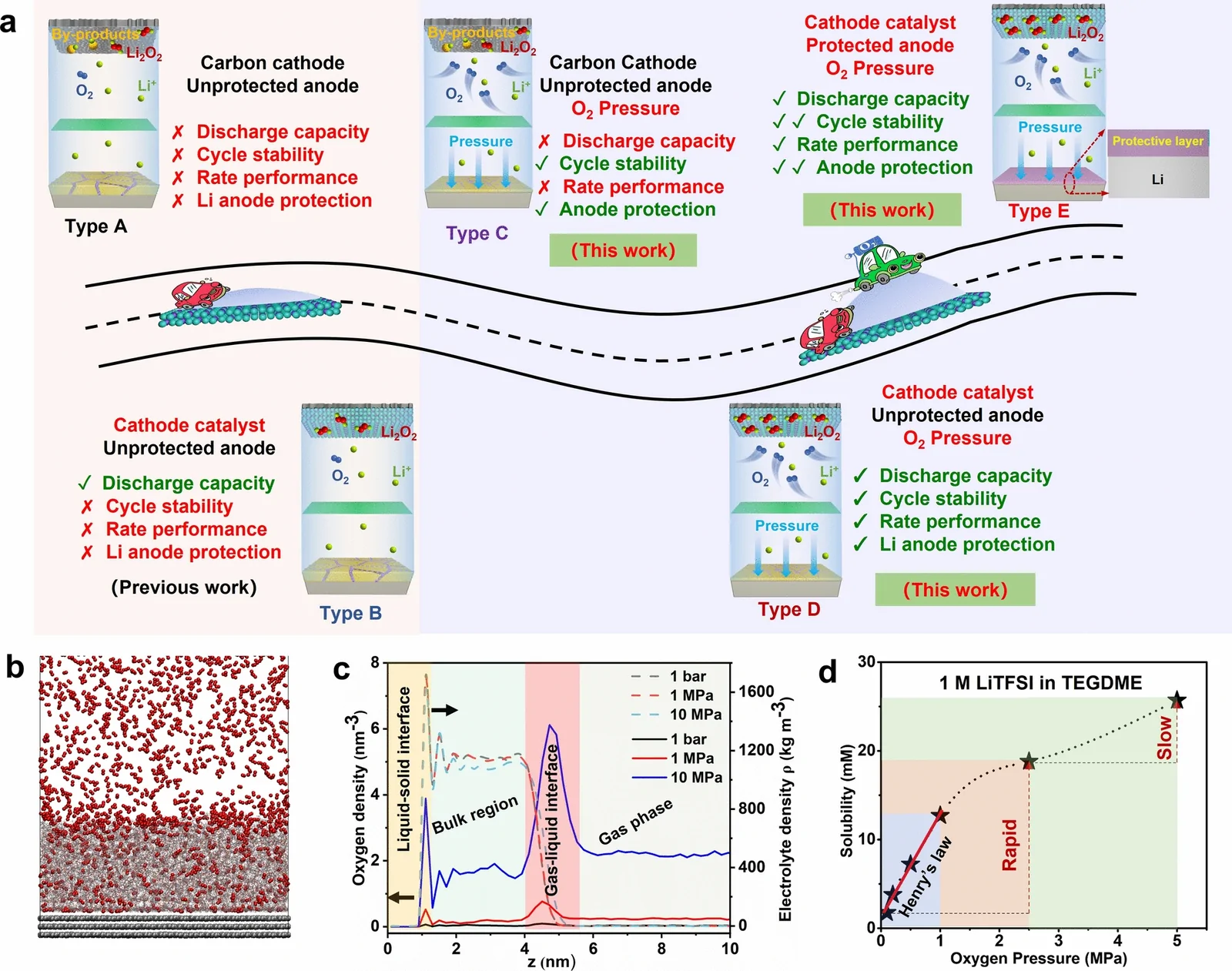
Conceptual design. **a** Developmental look at research on LOBs in this work. **b** Schematic of O_2_ dissolution in the electrolyte (1 M LiTFSI in TEGDME) under 10 MPa. **c** Density distributions of O_2_ molecules and electrolyte along the z axis. **d** Solubility curve of O_2_ in the electrolyte (1 M LiTFSI in TEGDME) under different O_2_ pressures

A conventional LOB (denoted as type A, Fig. 1a) is composed of an unprotected Li anode, an electrolyte and a carbon cathode. During operation, the high charge voltage easily induces electrolyte- and cathode-involved side reactions, resulting in limited cycle life of it. In response to this issue, cathode catalysts are usually introduced in LOBs (denoted as type B, Fig. 1a) to improve oxygen reduction reaction (ORR) and oxygen evolution reaction (OER) kinetics. However, poor O_2_ mass transport and charge transfer at high current densities evidently limit the formation and modulation of Li_2_O_2_. Increased charge potential is generally required to oxidize the insulating Li_2_O_2_ deposits, causing weak capacity retention and cycle stability at high rates. Therefore, an O_2_-pressure strategy was proposed with a hope to solve O_2_ mass transport and charge transfer simultaneously. The constructed O_2_-compensation LOBs are denoted as type C (carbon cathode), type D (catalyst cathode) and type E (catalyst cathode & protected Li anode, Fig. 1a), respectively, featuring the cells surrounded under a high-pressure O_2_ atmosphere.

Molecular dynamics (MD) simulation was employed to investigate the dissolution behaviour of O_2_ in the electrolyte under different O_2_ pressures. More O_2_ molecules are dissolved in the electrolyte under higher pressure (Figs. 1b and S1). The electrolyte density remains unchanged with the improvement of O_2_ pressure (Fig. 1c), while the O_2_ concentration significantly increases, especially at the liquid–solid interface, which is favourable for the overall promotion of ORR. On the basis of MD simulation, the solubility of O_2_ in the electrolyte was further measured under different pressures. Evidently, elevated O_2_ pressure effectively increases its solubility in the electrolyte (Fig. 1d), which can enhance O_2_ diffusion ability on the electrolyte-cathode interface to accelerate ORR kinetics expectantly [28–30]. Since Henry’s law applies to conditions of low solubility and low pressure, the solubility curve conforms to Henry’s law only in the range of 0.1–1 MPa. Additionally, O_2_-rich environment can induce the formation of Li_2-x_O_2_ with high conductivity at same limited discharge capacities compared with that in O_2_-poor condition, which is easily decomposed in the oxygen evolution reaction (OER) process to avoid accumulation of discharge products.

### KB-Based LOBs under High-Pressure O_2_

**Fig. 2:**
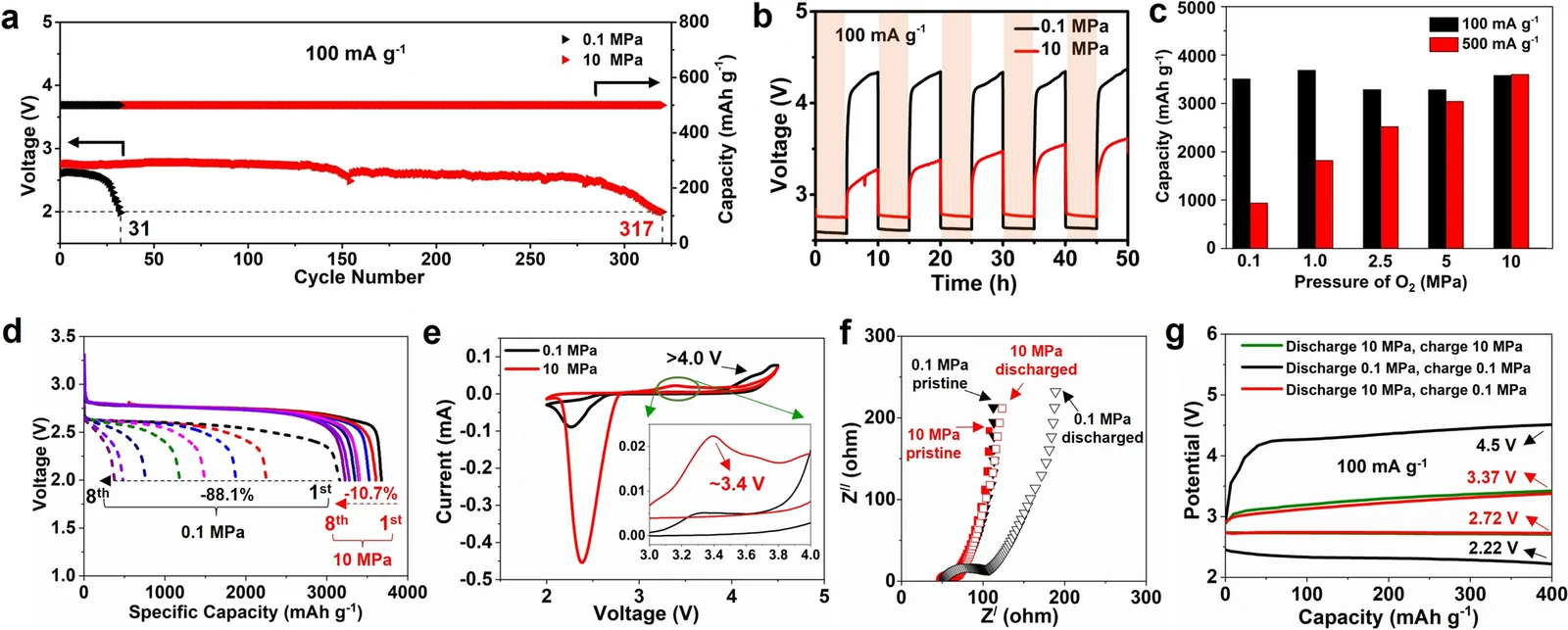
Electrochemical performance of KB-based LOBs. **a** Cycle performance and **b** discharge and charge voltages of KB-based LOBs at 100 mA g^−1^ with a limited capacity of 500 mAh g^−1^ under O_2_ pressure of 0.1 (type A) and 10 MPa (type C). **c** Comparison of discharge specific capacities of these batteries at 100 and 500 mA g^−1^ under different pressures. **d** Deep discharge capacities for the first 8 cycles at 100 mA g^−1^ and **e** cyclic voltammetry curves under 0.1 MPa and 10 MPa, respectively. **f** Electrochemical impedance spectroscopies of the pristine and discharged batteries under 0.1 MPa and 10 MPa, respectively. **g** Discharge and charge curves of batteries under different pressures

To exclude the interference from the catalyst factor, the high-pressure-O_2_ effect for optimizing cycle and rate performance was first investigated with traditional Ketjen Black (KB) carbon cathode independently (type C, Fig. 1a). The corresponding phase composition and morphology of KB are shown in Fig. S2. The type C battery operated under a O_2_ pressure of 10 MPa could continuously run 317 cycles (3,170 h) at 100 mA g^−1^ with a limited capacity of 500 mAh g^−1^ (Fig. 2a), which was much longer than that of type A battery (0.1 MPa, 31 cycles). This similar phenomenon was also verified at larger current density and in other carbon-based LOBs (Figs. S3 and S4). Long-life operation of type C battery is related with the remarkable decrease of overpotential under higher pressure (Figs. 2b and S5). It could realize higher round-trip efficiency and suppressed decomposition of the electrode materials and electrolyte. The rate performance under different pressures is further studied (Figs. 2c and S6). There is not obvious degradation of the discharge capacity at 100 mA g^−1^ under different pressures because the supply of O_2_ and the transfer of charges can meet the need of ORR at small current density. The difference of discharge capacities under 0.1–10 MPa is mainly derived from the slight difference of cathode loading mass. Because the coating process of KB cathodes is carried out manually, slight fluctuation of loading mass is normal. However, only a discharge capacity of ~ 27% can be maintained from 100 to 500 mA g^−1^ for the type A battery (Fig. 2c). In contrast, the difference of discharge capacities at 100 and 500 mA g^−1^ gradually fades away under higher O_2_ pressure. Sufficient capacity maintenance is realized for the type C battery under 5–10 MPa, which is over 3-fold increase compared with that for type A. It should be attributed to the accelerated reaction kinetics via enhanced O_2_ supply under high pressure but not the slight difference of cathode loading mass. The deep discharge capacities for the first 8 cycles were further tested under different pressures (Figs. 2d and S7). The discharge curves exhibit much smaller decay in capacity under 10 MPa. The ratio of capacity retention is over 89% even after 8 cycles, demonstrating superior deep discharge reversibility. Accordingly, O_2_ provision is the key step for the ORR process at high rates.

The cyclic voltammetry (CV) curves exhibit much higher onset discharge potential and larger peak current with respect to elevating O_2_ pressure (Figs. 2e and S8), significantly accelerating the catalytic activity, which results in forming defective discharge products Li_2-x_O_2_ with poor crystallinity. They are easily decomposed at a charge voltage of 3.4 V. In contrast, a potential over 4 V is required to decompose Li_2_O_2_ with high crystallinity formed under 0.1 MPa. Starting from type A (0.1 MPa) and type C (10 MPa) batteries with similar electrochemical impedance spectroscopies (EIS, Fig. 2f), the interfacial impedance of type C discharged to 500 mAh g^−1^ at 100 mA g^−1^ is much smaller than that of type A. It demonstrates the formation of discharge products with poor crystallinity and high conductivity under higher pressure, which is further confirmed by the X-ray diffraction (XRD, Fig. S9) and scanning electron microscope (SEM, Fig. S10) results. The wettability of electrolyte on the cathode under different pressure was also investigated. The contact angle of electrolyte on the KB cathode markedly decreases under higher O_2_ pressure (Fig. S11), demonstrating the improved wettability of electrolyte on the cathode, which is favorable for Li^+^ diffusion in the cathode and promotes reaction kinetics. Furthermore, a variable-pressure battery was operated (Fig. 2g), namely discharging under 10 MPa and recharging under 0.1 MPa, in comparison with constant-pressure batteries. Consequently, the initial discharge and charge profiles are overlapped with that under constant 10 MPa, indicating the discharge products formed under high pressure are more easily decomposed at lower charge potential. Accordingly, the much better performances demonstrate the key role of high O_2_ pressure for accelerating reaction kinetics of traditional LOBs.

### Catalyst-Based LOBs under High-Pressure O_2_

Instead of traditional carbon cathodes, a nitrogen-doped carbon-supported Co_3_Ru nanodots (NC/Co_3_Ru-NDs) catalyst obtained from our previous work (Fig. S12) [31], as a proof-of-concept, was assembled in the cathode to deeply investigate the O_2_-pressure effect for catalyst-based LOBs. Because the main purpose for KB-based batteries is to deeply study the effect of O_2_ pressure for the enhancement of electrochemical performances, there is no upper limit set for the pressure. As shown in Fig. 1d, with the increase of O_2_ pressure, the solubility of O_2_ also continuously increases, which provides the possibility for accelerating the reaction kinetics. The test results show that the electrochemical behaviors, including cycling property, rate performance and capacity retention rate, of the KB-based LOBs have been significantly improved with the increase of pressure. However, the O_2_ solubility and the corresponding test data also present that the electrochemical performance significantly improves from 0.1 to 2.5 MPa. With the pressure increased from 2.5 to 10 MPa, although the battery performance is continuously improved, the improvement efficiency has significantly slowed down. Therefore, in the catalyst-based LOBs, the comparison of high-pressure test is set at 2.5 MPa. In addition, compared with KB-based batteries, the reaction kinetics of catalyst-based batteries has already been significantly enhanced via catalyst cathodes, so the demand for the effect of pressure is relatively moderate. Meanwhile, from the perspective of battery practicality and safety, a relatively lower pressure condition is more meaningful. Accordingly, the preferred O_2_ pressure for type D battery is 2.5 MPa, which can also improve the wettability of electrolytes on the cathode under high-pressure O_2_ and be favorable for Li^+^ diffusion in cathodes (Fig. S13).

**Fig. 3:**
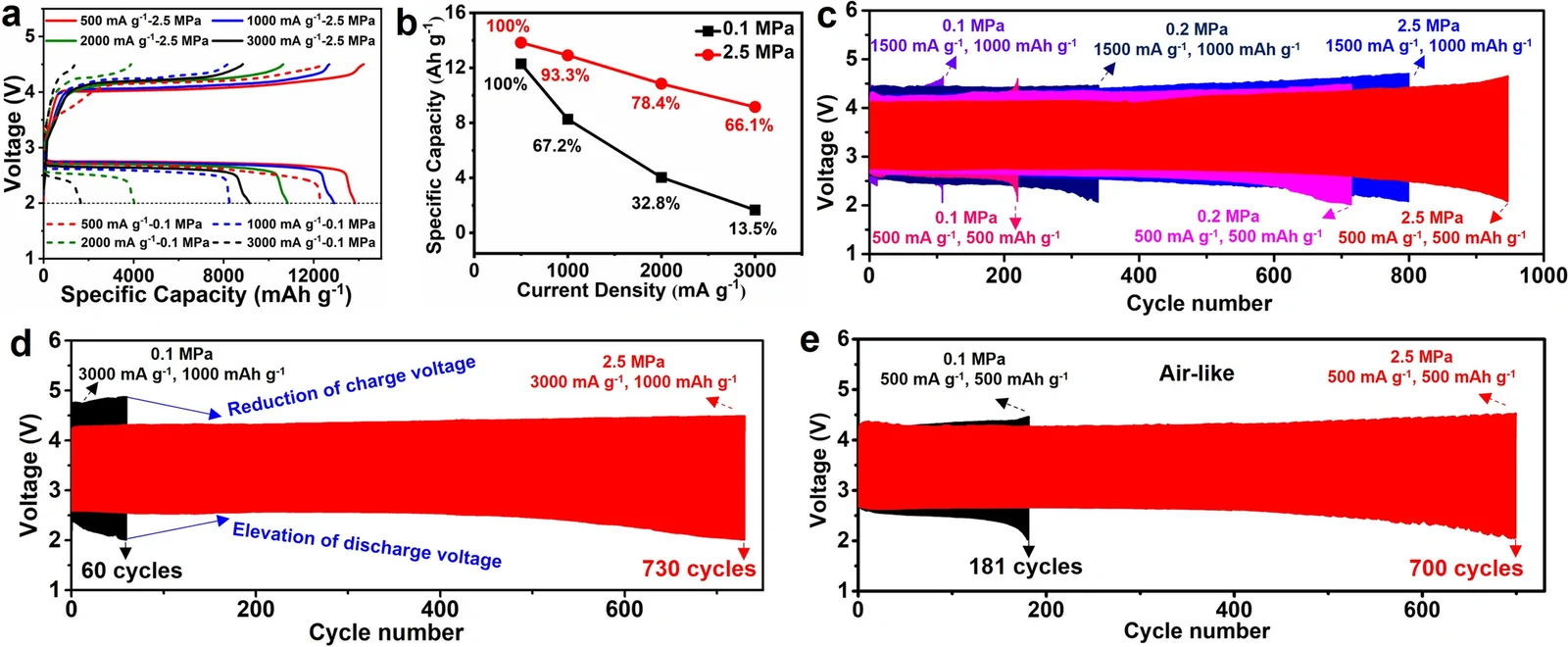
Electrochemical performance of catalyst-based LOBs**. a** Deep discharge–charge curves and **b** capacity retention rates of catalyst-based LOBs at 500–3,000 mA g^−1^ under 0.1 and 2.5 MPa. **c** Cycle performances of catalyst-based LOBs under 0.1, 0.2 and 2.5 MPa. Cycle performances of catalyst-based LOBs under 0.1 and 2.5 MPa **d** at 3,000 mA g^−1^ with a limited capacity of 1,000 mAh g^−1^ in O_2_ and **e** at 500 mA g^−1^ with a limited capacity of 500 mAh g^−1^ in air-like (industrial oxygen: 21%, industrial nitrogen: 79%) atmosphere

Although the type B battery displays a high discharge capacity over 12,000 mAh g^−1^ at 500 mA g^−1^, it dramatically declines to 1,655 mAh g^−1^ at 3,000 mA g^−1^ (Fig. 3a). In contrast, the type D battery exhibits a large capacity retention rate over 66% in the range of 500–3,000 mA g^−1^ (Fig. 3a, b). It is over five times as large (9,146 mAh g^−1^) at 3,000 mA g^−1^ compared with that under 0.1 MPa (Fig. 3a), which is an expected result of sufficient O_2_ supply. In the cycling process, when the environmental pressure is slightly raised from 0.1 to 0.2 MPa, the cycle life of batteries can markedly extend from 220 (type B) to 716 cycles at 500 mA g^−1^ (Figs. 3c and S14) [31]. Small-step improvement in O_2_ pressure realizes a big leap in battery cycle lifetime. More astoundingly, the type D battery operating under 2.5 MPa could run for 950 cycles (Fig. 3c), which is extended up to 4.3-fold compared with that of type B battery. When the current density and the limited specific capacity are increased to 1,500 mA g^−1^ and 1,000 mAh g^−1^, respectively, the type D battery could run for 800 cycles (Figs. 3c and S15)-more significant improvement than that of type B (109 cycles). Because more sufficient O_2_ provision is required at higher current density, the small-step improvement from 0.1 to 0.2 MPa could not meet the need of long-term stable cycle. These superior cycle performances are among the best reported in the absence of Li anode protection (Table S1).

Even at an extremely high current density of 3,000 mA g^−1^, the type D battery could steadily operate 730 cycles (Figs. 3d and S16), which is over 12-fold increase compared with that of the type B battery (60 cycles). The O_2_-pressure effect in raising cyclic stability is more prominent at higher current densities owing to the request of more O_2_ species. The former also exhibits raised discharge and reduced charge voltages, which is further confirmed by the CV results (Fig. S17). Actually, the accelerated reaction kinetics results in overpotential reduction at various current densities (500–3,000 mA g^−1^) under high-pressure O_2_ (Fig. S18). The leapfrog development in cyclic stability and rate capacity that are reproductive (Fig. S19) effectively conquers the repugnant issue of poor rate performance. Furthermore, pure O_2_ was replaced with air-like atmosphere to preliminarily explore the practical application. Fortunately, the battery could stably operate 700 cycles (~ 1,400 h) under 2.5 MPa (Figs. 3e and S20), which is more superior than that under 0.1 MPa. Accordingly, the pressure-compensation LOBs exhibit satisfied cyclic performance in both O_2_ and air-like environment under high pressure.

### O_2_-Compensation Mechanism for Redox Kinetics

**Fig. 4:**
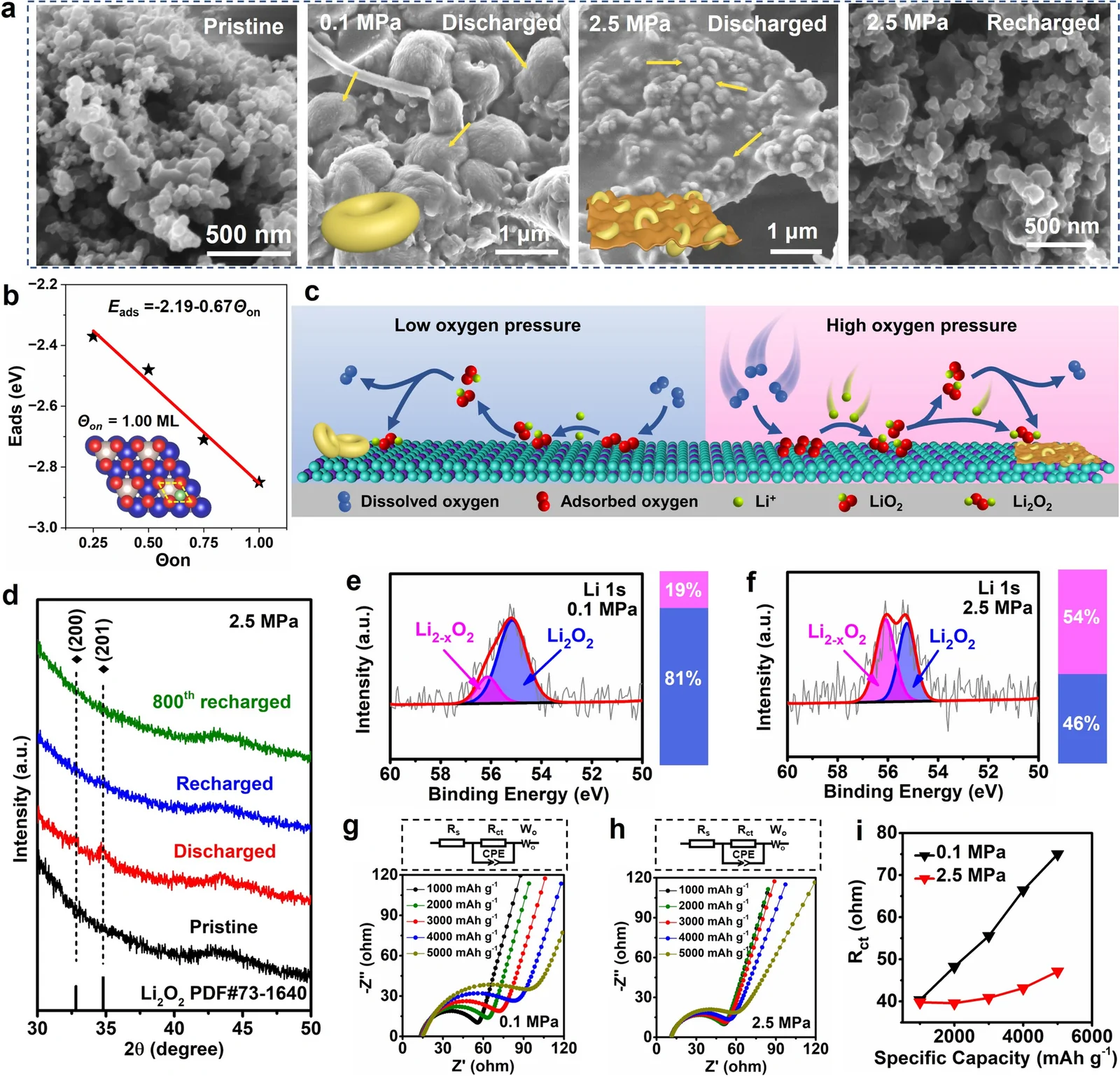
O_2_-Compensation Mechanism analysis. **a** SEM images of the cathodes: pristine, discharged to 5,000 mAh g^−1^ at 500 mA g^−1^ under 0.1 MPa and 2.5 MPa, and recharged under 2.5 MPa. **b** The relationship between *E*_ads_ of Li^+^ and *Θ*_on_ on Co_3_Ru (002) surface. **c** Schematic diagram of the formation mechanism of Li_2_O_2_ under low and high O_2_ pressures. **d** Ex-situ XRD patterns of the LOBs at different discharge/charge stages under 2.5 MPa. **e–f** High-resolution Li 1* s* XPS spectra of cathodes after discharged to 5,000 mAh g^−1^ at 500 mA g^−1^ under 0.1 and 2.5 MPa. **g–h** Electrochemical impedance spectroscopies and **i** *R*_ct_ values at different discharge stages at 500 mA g^−1^ under 0.1 MPa and 2.5 MPa. Insets in **g–h** are the corresponding equivalent circuit models

Deep analysis of discharge products is favorable for understanding the O_2_-compensation mechanism in tuning reaction kinetics and pathways. The discharge products have a typical toroidal morphology for type B (Fig. 4a), while films and small-sized toroidal particles are grown on the cathode of type D battery, indicating the formation of discharge products via dual growth pathways. Generally, the films and small-sized toroidal particles are generated at higher current density due to accelerated ORR process [4], which demonstrates the enhanced catalysis activity under the designed O_2_-compensation circumstance. The hybrid structure ensures strong interaction between discharge products and the cathode for efficient charge transfer, facilitating the decomposition of discharge products during charging. After recharged, the cathode recovers to its initial morphology. Accordingly, long-cycle and high discharge capacity are simultaneously achieved in the O_2_-compensation condition.

Density functional theory (DFT) calculation further demonstrates the O_2_-compensation effect on tuning the growth pathway of discharge products. Because more dissolved oxygen is enriched on the surface of catalyst with O_2_-pressure elevation, the difference of O_2_ pressure is indirectly expressed by oxygen coverage (*Θ*_on_) difference in calculation [32]. Four stable ordered surface phases with O (red ball) located on the (002) surface of Co_3_Ru catalyst are constructed (Figs. 4b and S21), corresponding to 0.25, 0.50, 0.75, and 1.00 monolayer (ML) *Θ*_on_, respectively. Evidently, high *Θ*_on_ could promote the formation of LiO_2_ intermediate (O_2_* + Li^+^  + e^−^ → LiO_2_), which accelerates the nucleation of discharge products by the “solution pathway” (LiO_2_ + LiO_2_ → Li_2_O_2_ + O_2_). Thus, it induces the formation of small-sized particles. Meanwhile, the improved *Θ*_on_ positively gives rise to the increased adsorption energy (*E*_ads_) of Li^+^ (green ball) on the catalyst (Fig. 4b). The relationship between *E*_ads_ and *Θ*_on_ conforms to a linear equation (*E*_ads_ = −2.19–0.67*Θ*_on_). This induces partial LiO_2_ converting into film-like discharge products by the “surface pathway” (LiO_2_ + Li^+^  + e^−^ → Li_2_O_2_), enhancing the interaction between discharge products and catalysts. Consequently, the hybrid structure is obtained by the dual growth pathways under high-pressure O_2_, schematically illustrated in Fig. 4c.

Some ex-situ measurements were further conducted. The XRD patterns show that Li_2_O_2_ is the main discharge products with poor crystallinity in type D batteries (Figs. 4d and S22), which could be decomposed adequately even after 800 cycles under 2.5 MPa. Although the catalyst cathode discharges at the same limited capacity under 0.1 and 2.5 MPa, the ratio of Li_2-x_O_2_ in discharge products show significant difference. The ratio of Li_2-x_O_2_ (56.2 eV) in discharge products is prominently improved from 19% (type B, Fig. 4e) to 54% (type D, Fig. 4f) [33, 34], indicating highly defective concentration of discharge products for the later, which is further verified via electron paramagnetic resonance (EPR) analysis. As shown in Fig. S23, the discharge products formed under 0.1 MPa exhibit a much weaker EPR signal than them under 2.5 MPa, confirming a higher concentration of Li vacancies for the later. Accordingly, the higher conductivity of discharge products formed under 2.5 MPa is demonstrated. Accordingly, the gap of charge-transfer resistance (*R*_*ct*_) between type B and D is more distensible with discharge from 1,000 to 5,000 mAh g^−1^ (Fig. 4g-i and Table S2). Because Li_2-x_O_2_ is more easily decomposed than Li_2_O_2_ during charging, the type D battery is more stable operation than type B. These results demonstrate improved reaction kinetics, optimized reaction pathways, decreased resistance, and suppressed accumulation of discharge products under high-pressure O_2_, achieving the purpose of long cycle life at high current densities.

### Suppressing Corrosion of Unprotected Li Anode by Pressure Effect

**Fig. 5:**
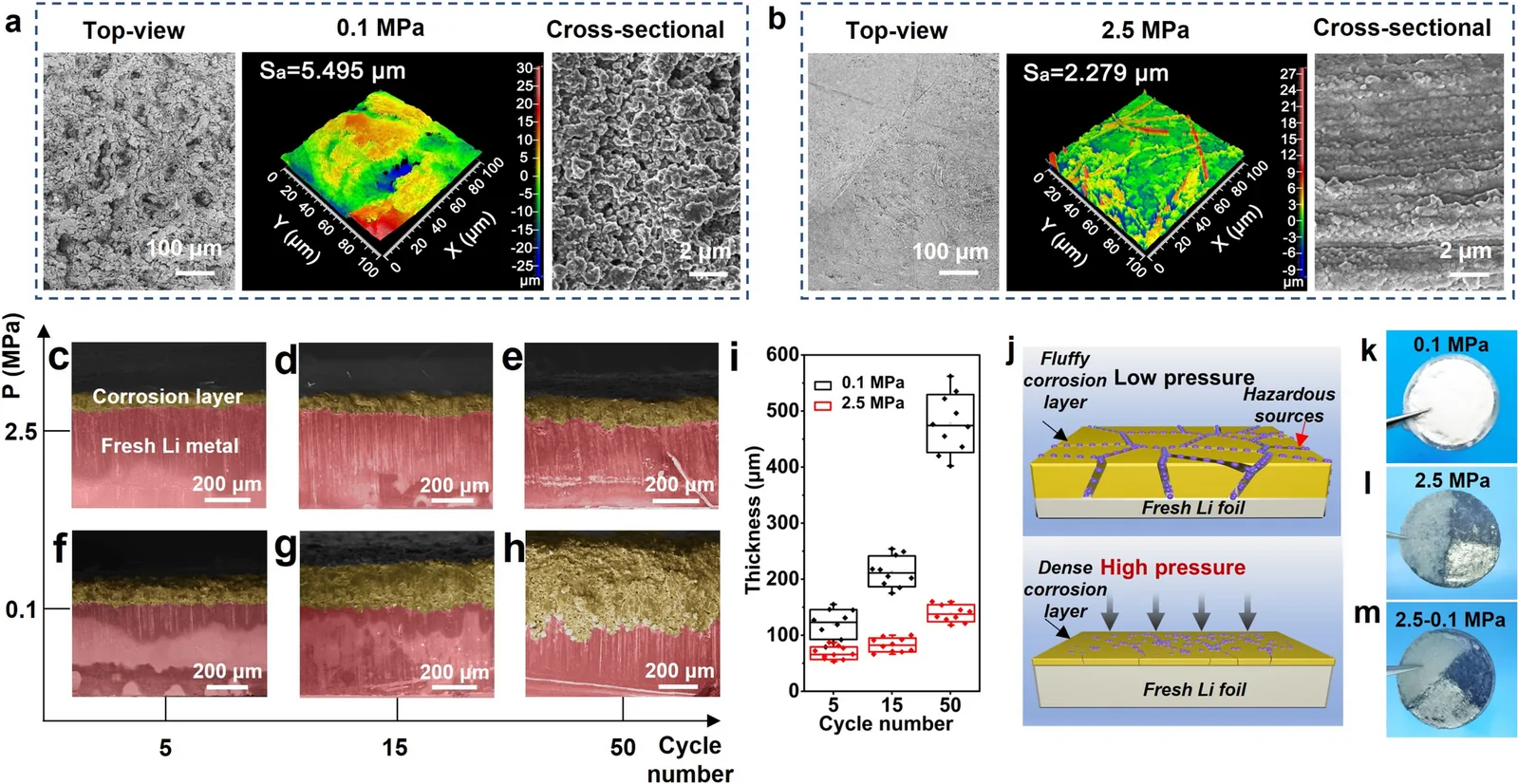
Top-view SEM, corresponding three-dimensional surface and cross-sectional SEM images of the corrosion layers after 5 cycles at 500 mA g^−1^ with a limited capacity of 500 mAh g^−1^ under **a** 0.1 MPa and **b** 2.5 MPa. Cross-sectional SEM images of anodes under 2.5 MPa after **c** 5 cycles, **d** 15 cycles, and **e** 50 cycles, and 0.1 MPa after **f** 5 cycles, **g** 15 cycles, and **h** 50 cycles at 500 mA g^−1^ with a limited capacity of 500 mAh g^−1^. **i** Box plot of the corrosion layer thickness at different cycles under 0.1 and 2.5 MPa. **j** Schematic illustration of the pressure effect on Li anodes protection. Optical images of Li anodes after batteries failure under **k** 0.1 MPa, **l** 2.5 MPa and **m** 2.5–0.1 MPa

Besides enhanced reaction kinetics, corrosion inhibition of unprotected Li anode also plays a critical role in superior stability of type D battery. Limiting the porosity of initially formed corrosion layers is one of the key issues in suppressing rapid corrosion of unprotected Li anodes. When LOBs are operated under high pressure, the gas pressure is transmitted to the surface of Li anodes through the gas–liquid-solid interface. Under the effect of vertical isostatic pressure, fortunately, a dense corrosion layer with high flatness (Fig. 5b) is formed in the type D battery instead of a fluffy structure (type B, Fig. 5a). The compactness of LiOH corrosion layer is positively correlated with the O_2_ pressure (Fig. S24), which could act as a contact barrier to inhibit the shuttle of corrosion sources. The chemical composition and relative content of the corrosion layer further show that escalated pressure plays a key role in delaying the corrosion rate of Li anodes (Fig. S25), which results in marked reduction in thickness of corrosion layers (Figs. 5c–h and S26). The gap of them (Fig. 5i) is further enlarged with the extension of cycle time because high pressure renders the initial corrosion surface denser and hinders the interaction between lithium atoms and corrosion sources. Consequently, the thickness of corrosion layer exhibits a marginal increment over time (Fig. 5c–e). This effect is independent of gas types (Fig. S27), which should be a general strategy in mitigating the corrosion of Li anodes (Fig. 5j). Meanwhile, the interface between the corrosion layer and the fresh Li is smoother under high pressure, facilitating uniform lithium deposition and suppressing the formation of dead lithium. The Li anode operated for 50 cycles in type B and D batteries, respectively, and a fresh Li wafer are reassembled into Li||Li symmetric batteries to investigate the charge transfer and Li^+^ diffusion kinetics of corrosion layers. The result demonstrates strong Li^+^ transport and charge-transfer kinetics of the cycled anode in type D battery (Fig. S28 and Table S3) due to remarkable thinning of corrosion layers. Generally, densification of corrosion layers obstructs Li^+^ transport channels. However, the obvious thinning of them not only offsets the side effect of densification, but also enhances Li^+^ transport. In addition, solid electrolyte interface (SEI) films are generally formed on the surface of bare Li anode when LOBs are stood in O_2_ chambers before electrochemical tests. They are mainly composed of Li_2_O (Fig. S29) and can protect the bare Li anode in the inital period. However, they are instead of LiOH corrosion layers after a couple of cycles (Fig. S25). Meanwhile, O_2_ pressure hardly affects the formation and composition of SEI films (Fig. S29). It further demonstrates the key role of the densification of corrosion layer for protecting bare Li anode but not SEI films.

After failure of type B and D batteries, these anodes were tested to illustrate the anticorrosion ability of them. The Li anode is completely converted into loose white LiOH in type B (220 cycles, Figs. 5k and S30), while partial Li metal in type D is still maintained even after 950 cycles (Fig. 5l). Visibly, the pressure strategy free of artificial protection layers achieves a breakthrough in anticorrosion of unprotected Li anodes. To further illustrate the effect of dense structure, a battery was first operated for 10 cycles under 2.5 MPa to preform a dense corrosion layer and then run till failure under 0.1 MPa. Resultantly, the battery could stably run for 539 cycles (Fig. S31). Meanwhile, the fresh Li metal partially remains after failure (Fig. 5m). The raised discharge voltage under high-pressure O_2_ is also observed in situ via the designed battery. This pressure-induced self-passivation of Li anodes via the preformation of dense corrosion layers is worth discussed in future.

### Ultralong Cycle Lifetime of LOBs under Dual-Strategy Effect

**Fig. 6:**
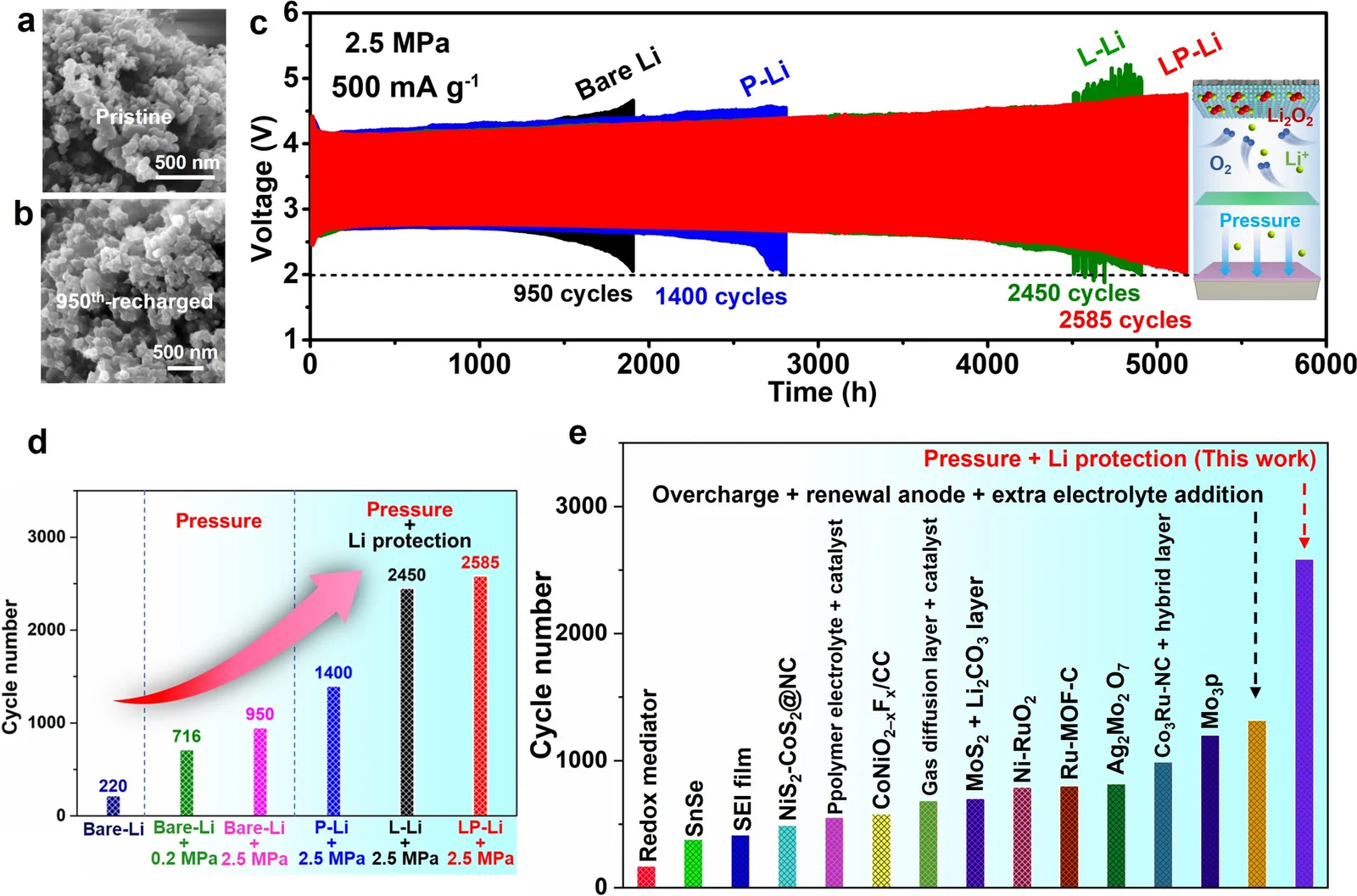
SEM images of **a** the pristine and **b** the 950th recharged cathodes under 2.5 MPa. **c** Cycle lifetimes of LOBs assembled with bare Li, P-Li, L-Li, and LP-Li anodes, respectively. **d** The optimizing process of different type LOBs in this work. **e** Comparison of cycle performance with reported works at similar current densities

The failure reason of type D batteries was investigated from the view of the cathode and the anode. Actually, the cathode in type D remains well after 950 cycles (Fig. 6a, b) and obvious residue of discharge products is not observed (Fig. S32), indicating negligible passivation of active sites during the long-term cycling. On the anode, although high pressure could slow down the corrosion rate of Li metal during cycling, the dense corrosion layer is hundreds of microns in thickness after 950 cycles (Fig. S33). Efficient Li^+^ diffusion is inhibited in this case, causing a sharp increase of impedance, which may be the key factor of type D failure. When the electrolyte is reinjected in the failed type D, disappointingly, the cell could only run for 40 cycles under 2.5 MPa (Fig. S34). It should be attributed to the anode coated with a thick and dense corrosion layer, impeding the infiltration of electrolyte and Li^+^ diffusion. After the failed type D was reassembled with a fresh Li anode and reinjected with electrolytes, the cell could operate > 1,800 h (> 900 cycles) under 2.5 MPa once again (Fig. S34), which is comparable with that in Fig. 3c. The recycle of core cathode catalysts reduces not only the cost of batteries, but also environmental pollution.

This inspires us to achieve ultralong cycle lifetime in LOBs by combining artificial protective layers of Li anodes with the pressure passivation-corrosion strategy. Referring to our previous work [30], three types of representative protection layers were constructed on the surface of Li anodes, respectively, including 1H,1H,2H,2H-perfluorodecyltrimethoxysilane (PFDTMS, organic layers, P-Li), LiF/Sn/Li_5_Sn_2_ (inorganic layers, L-Li), and LiF/Sn/Li_5_Sn_2_-PFDTMS (inorganic–organic hybrid layers, LP-Li). The dual-strategy effect of pressure and protection layers is a general strategy to achieve a leapfrog improvement in the cycle life of batteries. When they are cycled at 500 mA g^−1^ under 2.5 MPa, the batteries assembled with P-Li and L-Li anodes could operate 1,400 and 2,450 cycles before failure (Figs. 6c and S35), respectively, which are much superior to those with bare-Li anodes under 0.1 (Fig. 3c) and 2.5 MPa (Fig. 6c), and the protected Li anodes under 0.1 MPa [30]. As shown in Fig. 3c, the LOB with the bare-Li anode can only operate 220 cycles under 0.1 MPa. Meanwhile, the Li anode is completely corroded into LiOH (Fig. 5k). When the L-Li anode is assembled in the LOB instead of bare Li anode, the batteries can operate more stable. Partial Li metal in the L-Li anode is maintained after 600 cycles under 0.1 MPa (Fig. S36a), but severe corrosion of it still occurs. In contrast, the markedly improved anticorrosion behavior of L-Li anode under 2.5 MPa can be clearly observed in Fig. S36b, presenting uniform corrosion interface and suppressed corrosion rate even after 2,450 cycles. The significant extension of cycle life should be closely related with the enhanced corrosion resistance of Li anode under the dual-strategy effect of pressure and protection layers. Furthermore, when the battery is assembled with the LP-Li anode (type E, Fig. 1a), it can continuously operate 2,585 cycles (Fig. 6c) over 11-fold increase for 220 cycles (Fig. 3c). The duration is over 7 months and replicable during the cycling process (Fig. S37). Figure 6d summarizes the optimization process of cycle performance in different type LOBs. Compared with reported works at similar current density, the cycle performance of type E battery is among the best (Fig. 6e and Table S1).

Be virtue of the above results, the extraordinary performance of LOBs can be attributed to two critical factors: promoting reaction kinetics of cathodes and limiting corrosion of Li anodes via high O_2_ pressure. Firstly, the elevation of O_2_ pressure increases the solubility of O_2_ in the electrolyte. This can effectively enhance the mass transfer ability of O_2_ in LOBs, accelerating the reaction kinetics at cathodes. The higher capacity retention and longer cycling lifetime have been achieved due to sufficient supply of O_2_ under high pressure. Excellent rate performance demonstrates enhanced ORR reaction kinetics. The film-like and small-sized toroid-like discharge products are generally generated at a high current density. Interestingly, they are formed at a low current density under high pressure. It demonstrates the enhancement of ORR catalytic reaction kinetics under the O_2_-compensation circumstance. More importantly, the enriched oxygen environment can promote the formation of high conductivity and easily decomposable Li-vacancy discharge products, effectively reducing the overpotential during charging/discharging processes. This can limit the side reactions and the accumulation of discharge products, thereby significantly improving the cycle life of LOBs. Secondly, the effect of high-pressure gas can be transmitted through the gas–liquid-solid phase and eventually reach the corrosion layer on the surface of Li anode, thereby reducing the porosity of the corrosion layer and increasing its density. A corrosion layer with a smaller porosity can act as a barrier between the internal fresh metallic lithium and the external corrosion source, thereby slowing down the corrosion kinetics of Li anodes, which ensures long lifetime cycling of LOBs.

## Conclusions

In summary, we present a sustainable high-pressure O_2_ strategy for achieving efficient O_2_ mass transport and Li anode protection simultaneously. We demonstrate its application in Li-O_2_ battery with an ultralong cycle life and high capacity retention. The “killing two birds with one stone” strategy can first solve the key issue of urgent requirement for O_2_ mass transport at high current densities to accelerate reaction kinetics and optimize reaction pathway. Second, the high-pressure circumstance can densify the corrosion layer on Li anodes to inhibit the shuttle of corrosion sources. Under the dual-strategy effect of high-pressure O_2_ and artificial protection layers, the LOB actualizes a record-high lifetime of ~ 5,170 h (2,585 cycles) at 500 mA g^−1^ under constant operation. This strategy aligns with the growing need for greener energy storage solutions. The new insight will open avenues for designing cost-effective, high-performance Li-O_2_ and Li-air batteries for sustainable O_2_ redox-based energy storage technologies.

## Supplementary Information

Below is the link to the electronic supplementary material.

Supplementary file1 (DOCX 10054 KB)
