# Supplementary material for: Oxygen-Pressure Protocol Breaking Cycle Limit of Continuously Reversible Lithium-Oxygen Batteries
Source: Nanomicro Lett. 2026 Jan 5;18:156. doi: 10.1007/s40820-025-01990-z (PMC12765789; doi:10.1007/s40820-025-01990-z)
Supplement: Supplementary file 1 — Supplementary file1 (DOCX 10054 KB) [file 40820_2025_1990_MOESM1_ESM.docx]

Supporting Information for

**Oxygen-Pressure Protocol Breaking Cycle Limit of Continuously Reversible Lithium-Oxygen Batteries**

Xinhang Cui^1,3, #^, Fenglong Xiao^1, #^, Guoliang Zhang^2^, Zhangliu Tian ^3^, Qingshan Bao^1^, Yanlu Li^1^, Deliang Cui^1,^*, Qilong Wang^4^, Feng Dang^2,^*, Wei Chen^3^, Haohai Yu^1,^*, Huaijin Zhang^1^ and Gang Lian^1,^*

^1^ State Key Laboratory of Crystal Materials, Shandong University, Jinan 250100, P. R. China

^2^ School of Materials Science & Engineering, Shandong University, Jinan 250061, P. R. China

^3^ Department of Physics, National University of Singapore, 2 Science Drive 3, 117543, Singapore

^4^ Key Laboratory for Special Functional Aggregated Materials of Education Ministry, School of Chemistry & Chemical Engineering, Shandong University, Jinan 250100, P. R. China

*^#^*Xinhang Cui and Fenglong Xiao contributed equual to this work.

*Corresponding authors. E-mail: [liangang@sdu.edu.cn](mailto:liangang@sdu.edu.cn) (Gang Lian); [haohaiyu@sdu.edu.cn](mailto:haohaiyu@sdu.edu.cn) (Haohai Yu); [cuidl@sdu.edu.cn](mailto:cuidl@sdu.edu.cn) (Deliang Cui); [dangfeng@sdu.edu.cn](mailto:dangfeng@sdu.edu.cn) (Feng Dang)

**S1 Experimental Section**

**S1.1 Chemicals and Materials**

Ruthenium (III) chloride hydrate (RuCl_3_·xH_2_O), Cobalt (II) chloride hexahydrate (CoCl_2_·6H_2_O), N-Methyl-2-pyrrolidone (NMP) and anhydrous ethanol (C_2_H_5_OH) were purchased from Aladdin Corporation. Glucose (C_6_H_12_O_6_) and Urea (CO(NH_2_)_2_) were purchased from Sinopharm Chemical Reagent Co., Ltd. Anhydrous tetraethylene glycol dimethyl ether (TEGDME) and lithium bis(trifluoromethane) sulfonamide (LiTFSI) were purchased from Suzhou Dodochem, China. Glass microfiber filters (GF/B, Whatman), Polyvinylidene fluoride (PVDF, Arkema) and Ketjen Black (KB, Lion Corporation) were purchased from other agents in China.

**S1.2 Synthesis of catalysts**

Typically, 0.2 mmol ruthenium chloride (RuCl_3_), 0.2 mmol cobalt chloride (CoCl_2_·6H_2_O), 33.3 mmol glucose (C_6_H_12_O_6_) and 33.3 mmol urea (CO(NH_2_)_2_) were added in 10 ml deionized water to form a homogeneous solution. A hydrothermal reaction was proceeded at 150 °C for 10 h. After that, the precursor was obtained by washing the as-prepared powders with anhydrous ethanol and deionized water, and drying them at 80 °C for 24 h. The precursor was then annealed at 900 °C under Ar for 2 h. Finally, the NC/Co_3_Ru-NDs sample was obtained.

**S1.3 Li anodes protection**

**1. Organic protection layer**

PFDTMS (volume) (0.5%) was added to the electrolyte (1.0 M LiTFSI/TEGDME) and stirred to homogenize. Bare Li was used as the anode, and then the organic protection layer was formed during battery operation.

**2. Inorganic protection layer**

Fresh Li foils were immersed into 20 mM SnF_2_-DMSO organic solution for 1 min to form LiF/Sn/Li_5_Sn_2_ inorganic layer. Then, the Li foils with LiF/Sn/Li_5_Sn_2_ inorganic layer were cleaned with DMSO and 1, 2-Dimethoxyethane (DME) solvents in turn to remove the residual SnF_2_ and impurities for several times. Finally, L-Li could be obtained after drying at 90 °C for 24 h. The whole procedures were conducted in a glove box filled with Ar (H_2_O < 0.01 ppm, O_2_ < 0.01 ppm).

**3. Inorganic-organic hybrid protection layer**

PFDTMS (volume) (0.5%) was added to the electrolyte (1.0 M LiTFSI/TEGDME) and stirred to homogenize. L-Li was used as the anode, and then the inorganic-organic hybrid protection layer was formed during battery operation.

**S1.4 Electrochemical Performance Measurements**

**1. Preparation of KB cathodes**

KB was mixed with PVDF in NMP with mass ratio of 8:2. The mixture is then dispersed in NMP and continuous stirring was applied for 12h for the well-dispersed slurry. Then the prepared slurry was coated on carbon paper (TORAY, TGP-H-060, hydrophobic). The as-prepared cathode was heat-dried in vacuum at 110 ^o^C for 12 h.

**2. Preparation of catalyst cathodes**

The cathode slurry was prepared by mixing catalyst (40 wt%), KB (40 wt%) and PVDF (20 wt%) into NMP. The slurry was uniformly coated on carbon paper to make the cathode and then heat-dried in vacuum at 110 °C for 12 h.

**3. Battery assembly**

The Li-O_2_ batteries consist of an oxygen cathode, a fresh Li foil anode or protective Li anode, and a glass fiber separator dipped in a 1.0 M LiTFSI/TEGDME electrolyte. The batteries were assembled in a glove box filled with argon (H_2_O < 0.01 ppm, O_2_ < 0.01 ppm). To guarantee the constant high-pressure environment, all tests were performed in well-sealed specially designed chambers.

**4. Electrochemical testing**

The LAND multi-channel battery tester (Wuhan Land Electronic Co., Ltd) operated the galvanostatic discharge/charge tests of the Li-O_2_ batteries. The current density and specific capacity were normalized by the calculated mass of catalyst. Cyclic voltammetry (CV) scanning was carried out on an electrochemical workstation (Chenhua, Shanghai, CHI760E) with a voltage range of 2.0-4.5 V. Electrochemical impedance spectroscopy (EIS) was also performed on this electrochemical workstation.

**S1.5 Material Characterizations**

Morphology images were acquired from field-emission scanning electron microscope (G300, Carl Zeiss) and white light interferometer (ZeGage Pro HR, ZYGO). X-ray diffractometer (SmartLab 9 Kw, Rigaku) was employed for crystal phase analysis. The surface element composition and combination states of catalysts were studied by X-‍Ray Photoelectron Spectroscopy (AXIS Supra, Kratos). Contact angles were collected by contact angle goniometer (JC2000D, Powereach). Raman spectra were recorded by Confocal Raman Microscope (DXR, Thermo−Fisher Scientific) with an excitation line of 633 nm. Fourier transformation infrared absorption spectra of the samples were recorded by using a Nicolet NEXUS 670 Fourier transformation infrared spectrometer, with a wavenumber resolution of 4 cm^-1^ (4000-650 cm^-1^).

**S1.6 Solubility of Oxygen in Electrolyte Measurement**

At room temperature (298K), the electrolyte (1.0 M LiTFSI in TEGDME) was sealed in a balloon, and then further placed into a high-pressure chamber containing thumbtacks on one side of the interior, which was connected to a high-precision gas pressure sensor. The air in the high-pressure chamber was replaced by pure oxygen, and then cut off the oxygen supply after pressurizing to the required pressure. Shake the high-pressure chamber so that the balloon was punctured by the thumbtack, and the electrolyte was exposed to oxygen. After standing for a period of time, the pressure in the high-pressure chamber would drop due to the dissolution of oxygen in the electrolyte. Record the pressure value displayed by the pressure sensor at this time. The amount of substance of oxygen could be obtained by the ideal gas law:

$PV=nRT$ (S1)

where *P* is the absolute pressure of oxygen, *V* is the volume of oxygen, *n* is the amount of substance of oxygen, *R* is the gas constant (8.314 J·mol^-1^·K^−1^) and *T* is the thermodynamic temperature.

The change of amount of substance of oxygen was defined as:

$\Delta n=n_{1}-n_{2}$ (S2)

where the *n_1_* represents the amount of substance of oxygen before dissolution, and *n_2_* represents the amount of substance of oxygen after dissolution.

The solubility of oxygen was calculated by:

$c=\Delta n/L$ (S3)

where *c* is the solubility of oxygen and *L* is the volume of electrolyte.

**S1.7 Computational Methods**

Molecular dynamics (MD) simulations were used to examine the solubility of O_2_ in electrolytes and distribution of O_2_ at interfaces under different pressure. They were performed in GROMACS using the General Amber Force Field (GAFF). The MD model is constructed by three areas, including the graphite electrode (solid) cut from the hexagonal structure in the direction of (002), the electrolyte (liquid) and O_2_ atmosphere (gas). The graphite slab is built by three layers of perfect graphite (002) facet. The electrolyte at a concentration of 1M LiTFSI in TEGDME comprises a simulation area of 10 nm × 10 nm × 5 nm. Different amounts of O_2_ molecules are employed as the gas phase (0.1-10 MPa). Topology files, and bonded and Lennard-Jones parameters were generated by using the AuToFF while the RESP atomic charges from Multiwfn3.8 program were used [S1]. The cutoff for the Lennard-Jones potential was set to 10 Å. The long-range Coulombic interactions were counted by a particle-particle particle-mesh. The initial periodic systems were set up using PACKMOL [S2]. The solid and liquid phases of the graphite and electrolyte were simulated by 5 ns NPT to obtain the equilibrium state. To quantitatively and comparatively analyze the dissolution behavior of O_2_ molecules, 100, 400, and 2000 O_2_ molecules were employed as the gas phase of 0.1, 1 and 10 MPa systems, respectively. Finally, the solid-liquid-gas three-phase simulation was continued for 20 ns with NVT ensemble and data collected. Only the last 5-ns trajectory was sampled for the analysis of final data.

All first-principles density functional theory (DFT) calculations were performed using the Vienna Ab Initio Simulation Package (VASP) [S3]. The core−valence interaction was described by the projector-augmented wave (PAW) method [S4], and the generalized gradient approximation of Perdew−Burke−Ernzerhof (GGA-PBE) with Grimme’s semiempirical DFT-D3 dispersion correction to account for the van der Waals (vdW) interactions was used [S5]. After the convergence test, the cut-off energy for the plane wave basis was set to 500 eV, the Brillouin zone was sampled by 5×5×1 k-points using the Monkhorst-Pack scheme. A Co_3_Ru (002) substrate model was constructed from a 2×2 surface unit cell and a vacuum region of more than 15 Å. The convergence criteria for residual force and energy were set to 0.02 eV Å^-1^ and 10^-6^ eV, respectively. The adsorption energy (*ΔE*_ads_) of the adsorbates was obtained as

${\Delta E}_{ads}=E_{total}-E_{substrate}-E_{adsorbate}$ (S4)

where *E*_substrate_ is the energy of the Co_3_Ru (002) surface with different oxygen coverages (*Θ*_on_), *E*_adsorbate_ is the energy of the individual adsorbed species (Li^+^) and *E*_total_ is the total energy of the optimized substrate with adsorbate.

**S2 Supplementary Figures and Tables**

**
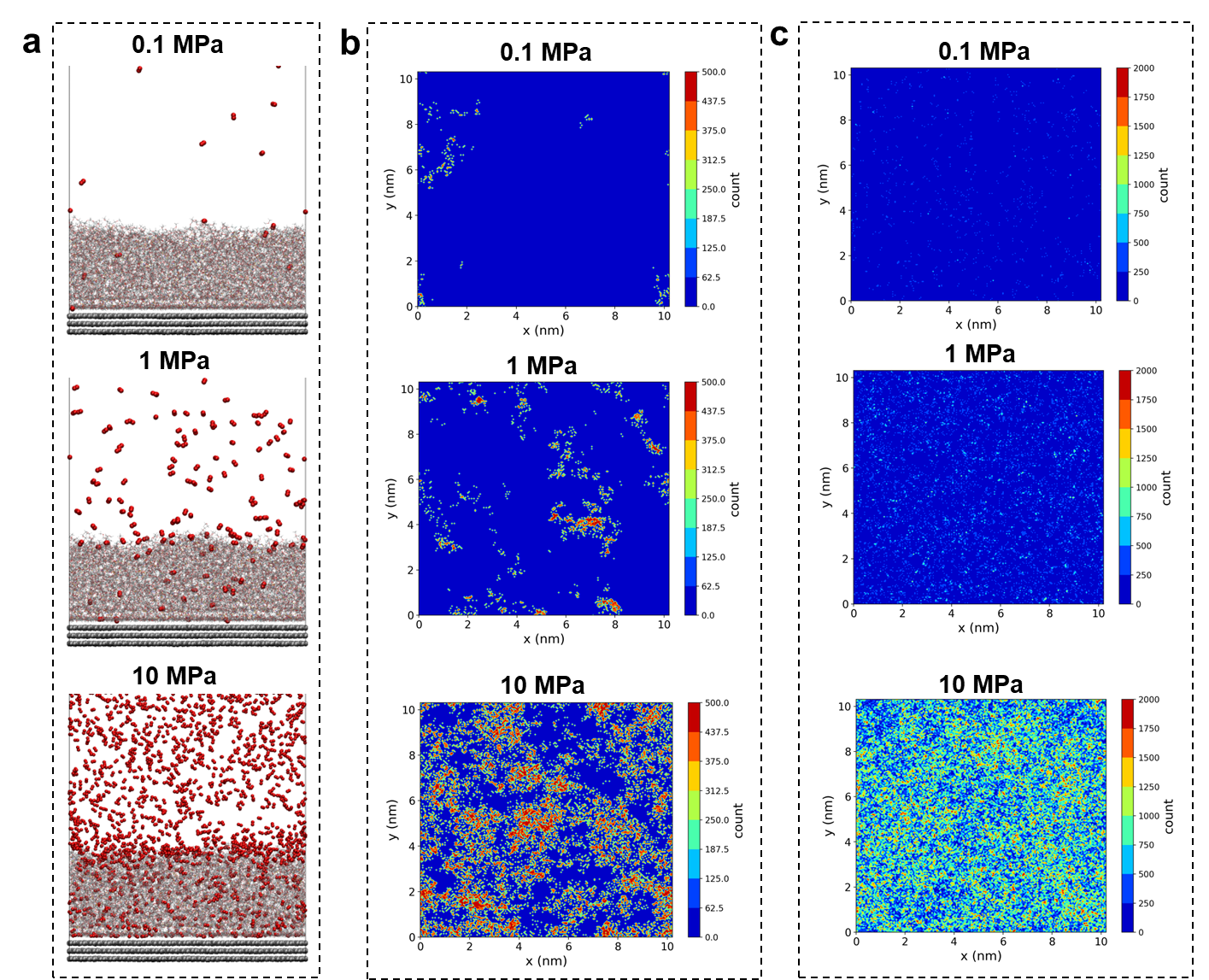
**

**Fig. S1** Molecular dynamics simulation of dissolution behavior of O_2_ in the electrolyte (1 M LiTFSI in TEGDME). (**a**) Schematic of O_2_ dissolution in the electrolyte under different O_2_ pressures. Number distributions of O_2_ molecules through the (**b**) gas-liquid and (**c**) gas-liquid interfaces under different pressures

The MD model is constructed by three areas, including the graphite (002) slab, the electrolyte comprising an area of 10 nm x 10 nm x 5 nm, and O_2_ atmosphere. To comparatively analyze the dissolution behavior of O_2_, 100, 400, and 2000 O_2_ molecules are inserted into the vacuum region above the electrolyte as the gas phase of 0.1, 1 and 10 MPa, respectively. The number distributions of O_2_ molecules through the x-y planes of these interfaces are examined to further illustrate the dissolution and permeation properties of O_2_ molecules.


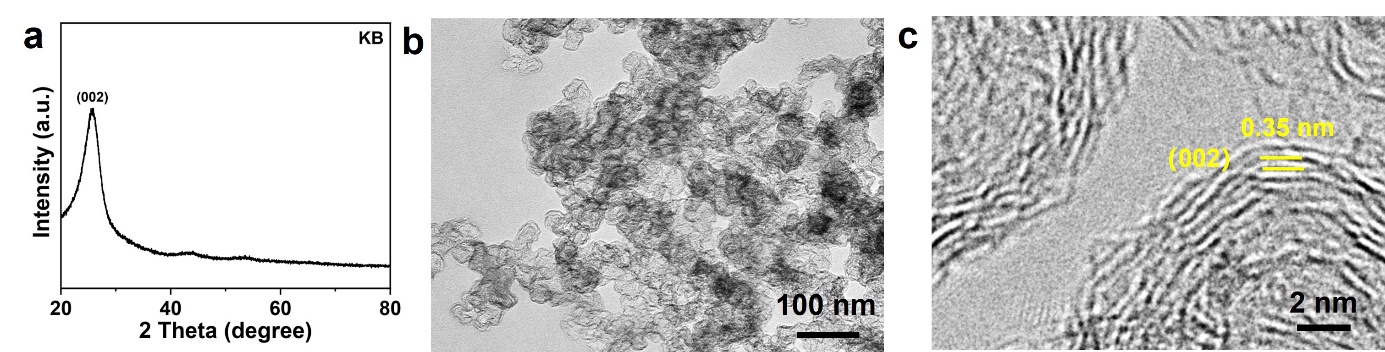


**Fig. S2** (**a**) XRD pattern, (**b**) TEM image and (**c**) HRTEM image of KB

The phase composition and microstructure of KB have been measured. The XRD pattern shows that this sample is composed of pure carbon material. The peak at ~25.6° corresponds to the (002) plane of graphite. The TEM image shows that the particle size of KB is uniform. Clear lattice fringes can be observed in the HRTEM image. The interlayer spacing is 0.35 nm, corresponding to the (002) plane of graphite.


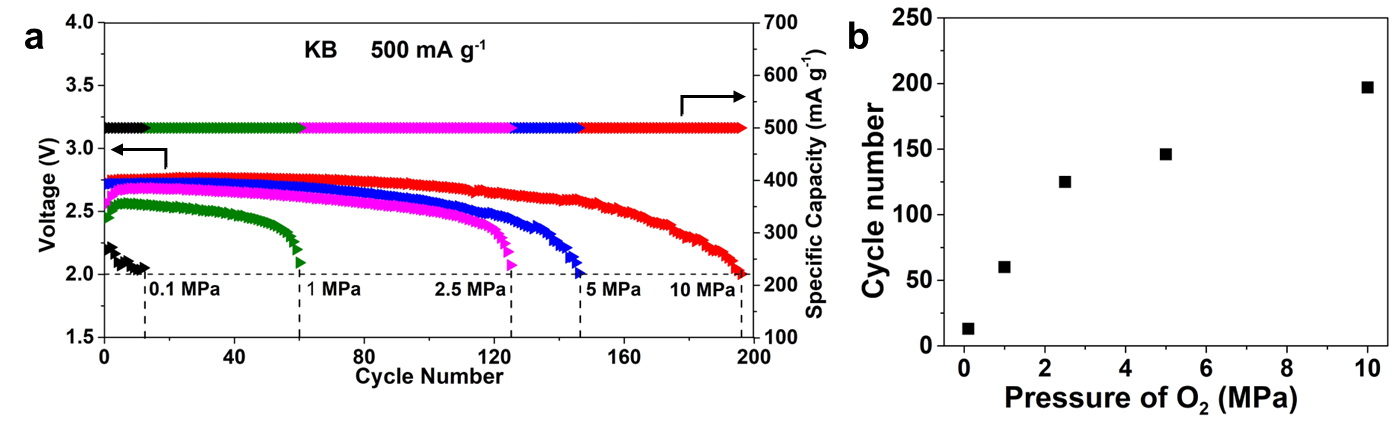


**Fig. S3** (**a**) Cycle lifetimes of KB-based LOBs at 500 mA g^-1^ with a limited capacity of 500 mAh g^-1^ under different O_2_ pressures. (**b**) The relationship between pressure of O_2_ and cycle number.

Rapid promotion of the cycle life could be achieved from 0.1 to 2.5 MPa, which gradually tends to moderation from 2.5 to 10 MPa. This tendency is consistent with the improvement of O_2_ solubility under different pressures.

**
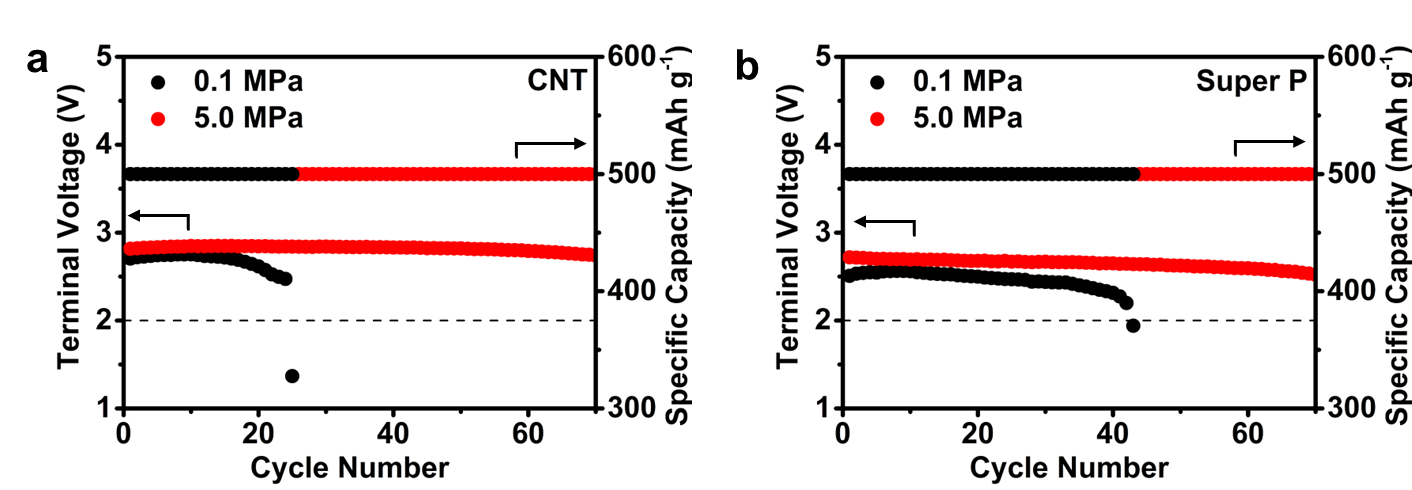
**

**Fig. S4** Cycle stabilities of (**a**) CNT- and (**b**) super P-based LOBs under different O_2_ pressure at a current density of 500 mA g^-1^ with a limited capacity of 500 mAh g^-1^

~~
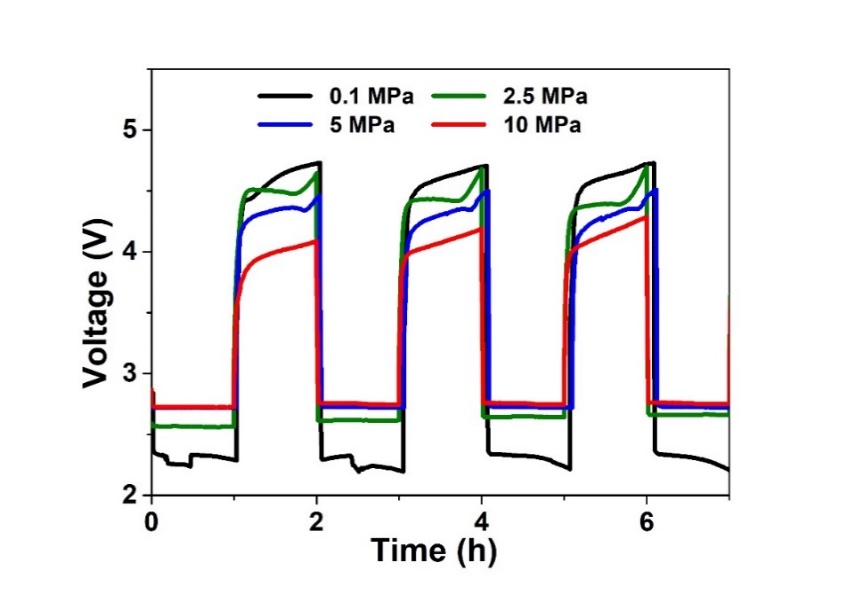
~~

**Fig. S5** Discharge and charge voltages of KB-based LOBs at 500 mA g^-1^ with a limited discharge capacity of 500 mAh g^-1^ under different pressure of O_2_


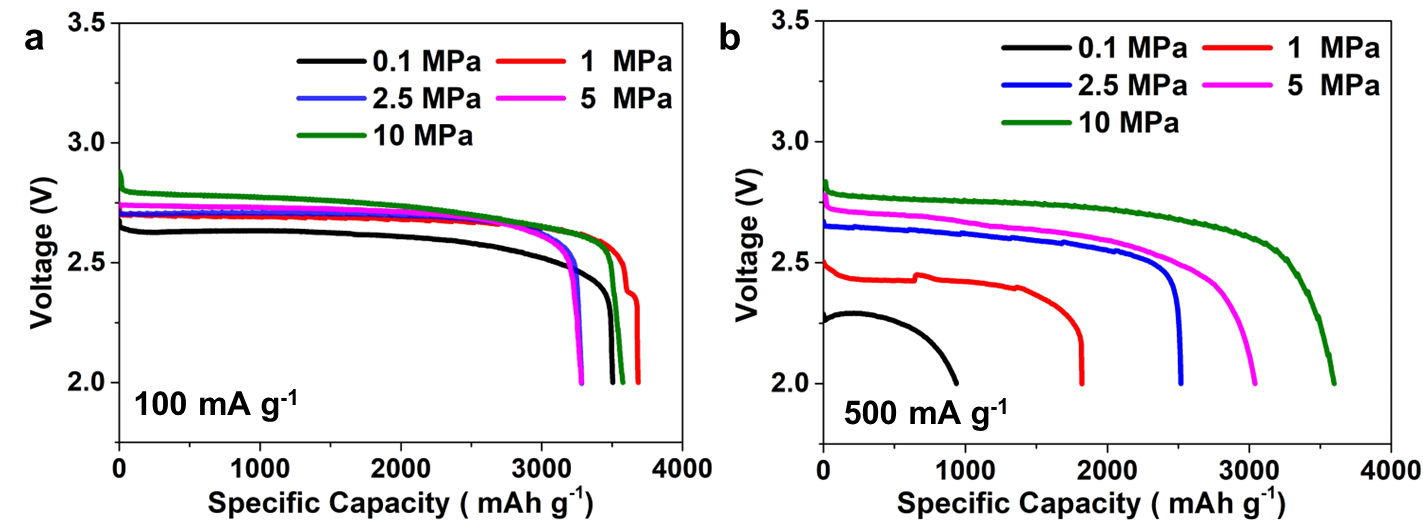


**Fig. S6** Discharge specific capacities of KB-based batteries at (**a**) 100 and (**b**) 500 mA g^-1^ under different pressure


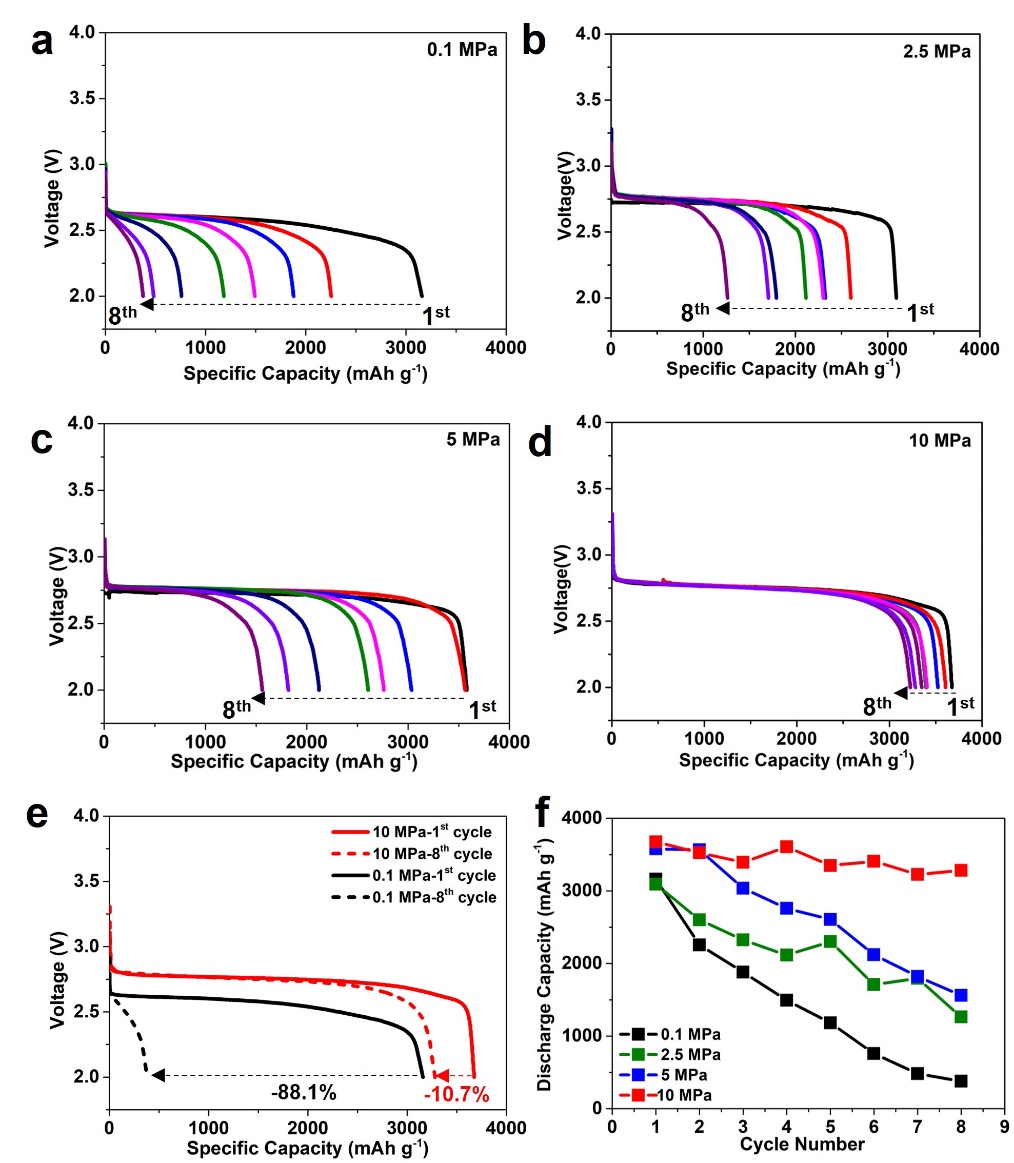


**Fig. S7** Deep discharge capacities from the 1^st^-8^th^ cycles at 100 mA g^-1^ under (**a**) 0.1, (**b**) 2.5, (**c**) 5 and (**d**) 10 MPa. (**e**) The comparison of deep discharge capacities of the 1^st^ and 8^th^ cycles under 0.1 and 10 MPa, respectively. (**f**) The comparison of deep discharge capacities from the 1^st^ to 8^th^ cycles at 100 mA g^-1^ under 0.1, 2.5, 5 and 10 MPa


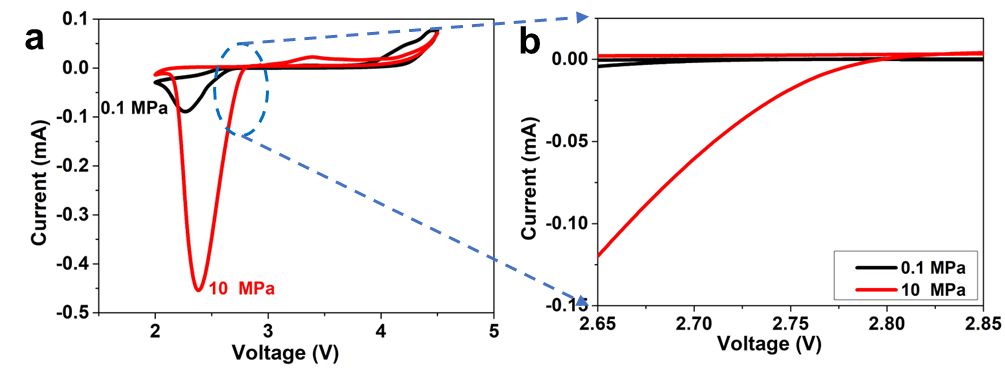


**Fig. S8** (**a**) CV curves of the KB-based LOBs under 0.1 (type A) and 10 MPa (type C) with a voltage window of 2.0-4.5 V. (**b**) Corresponding magnified area in (a)

**
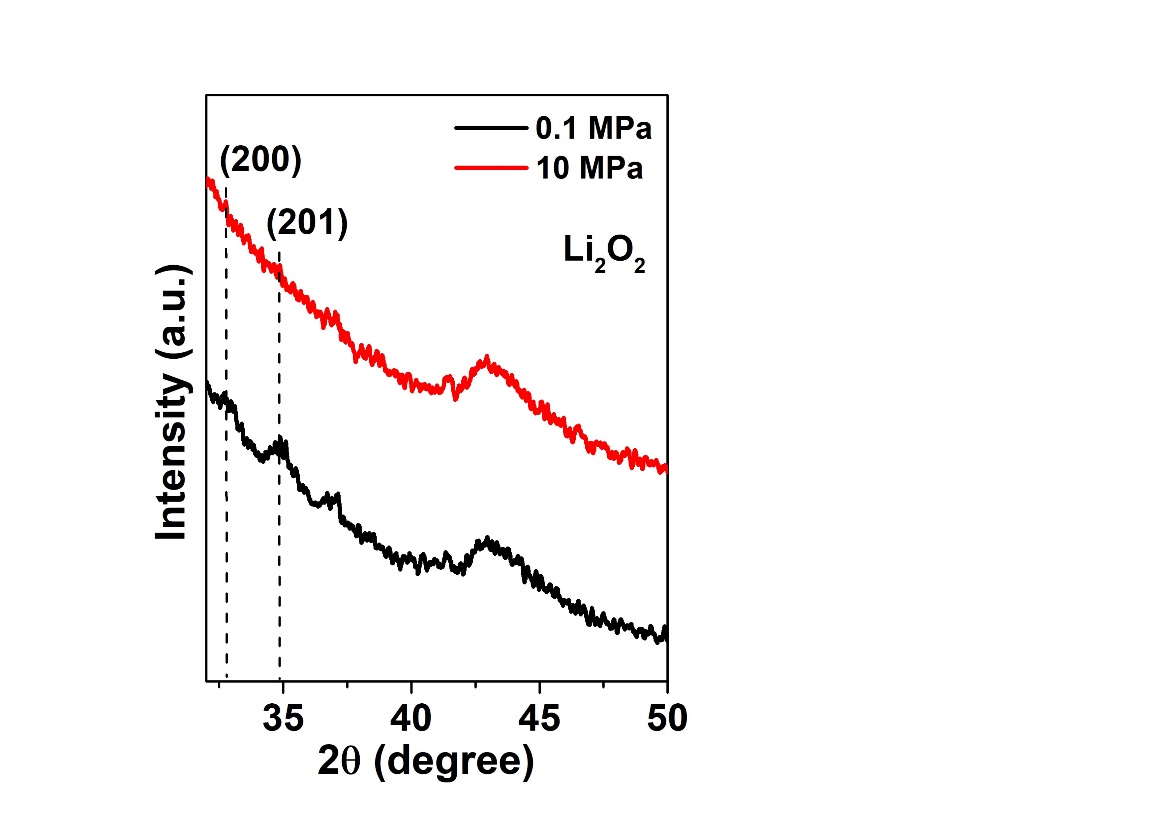
**

**Fig. S9** XRD patterns for discharged cathodes at 100 mA g^-1^ with a limited specific capacity of 500 mAh g^-1^ under 0.1 and 10 MPa, respectively

The peaks at ~32.7° and ~34.8° correspond to (200) and (201) planes of Li_2_O_2_.

**
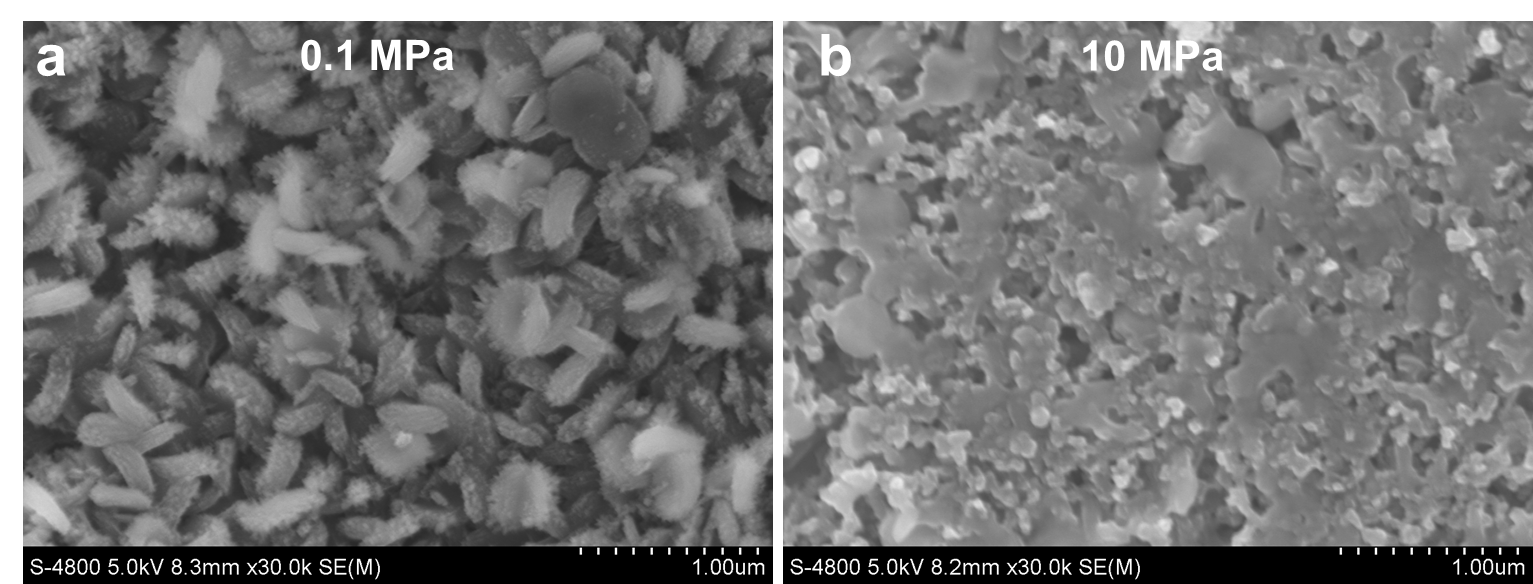
**

**Fig. S10.** SEM images of the cathodes discharged to 1000 mAh g^-1^ at 500 mA g^-1^ under (**a**) 0.1 MPa and (**b**) 10 MPa

Usually, the film-like discharge products Li_2_O_2_ is rich in the Li-vacancy. Thus, the formed amorphous Li_2-x_O_2_ film under high pressure is more conductive than the particle-like discharge products with high crystallinity.


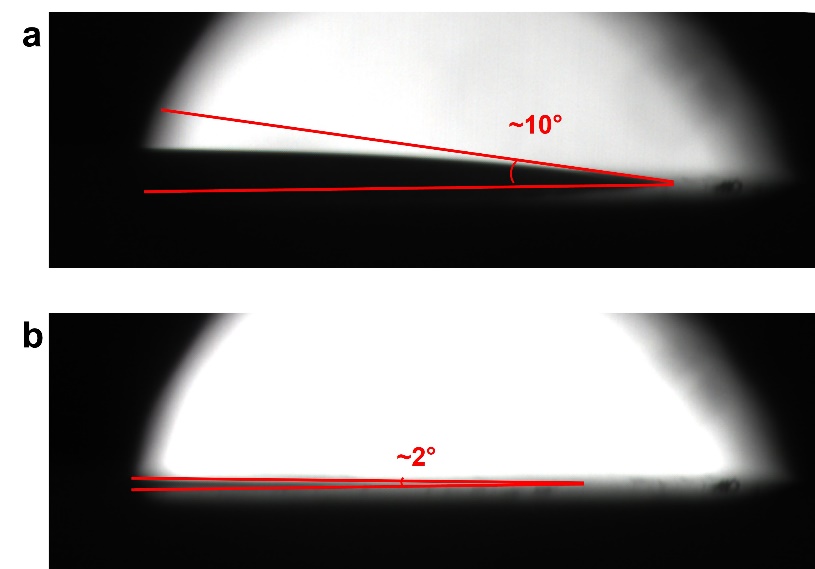


**Fig. S11** Contact angles between the electrolyte and the KB cathode under (**a**) 0.1 and (**b**) 10 MPa


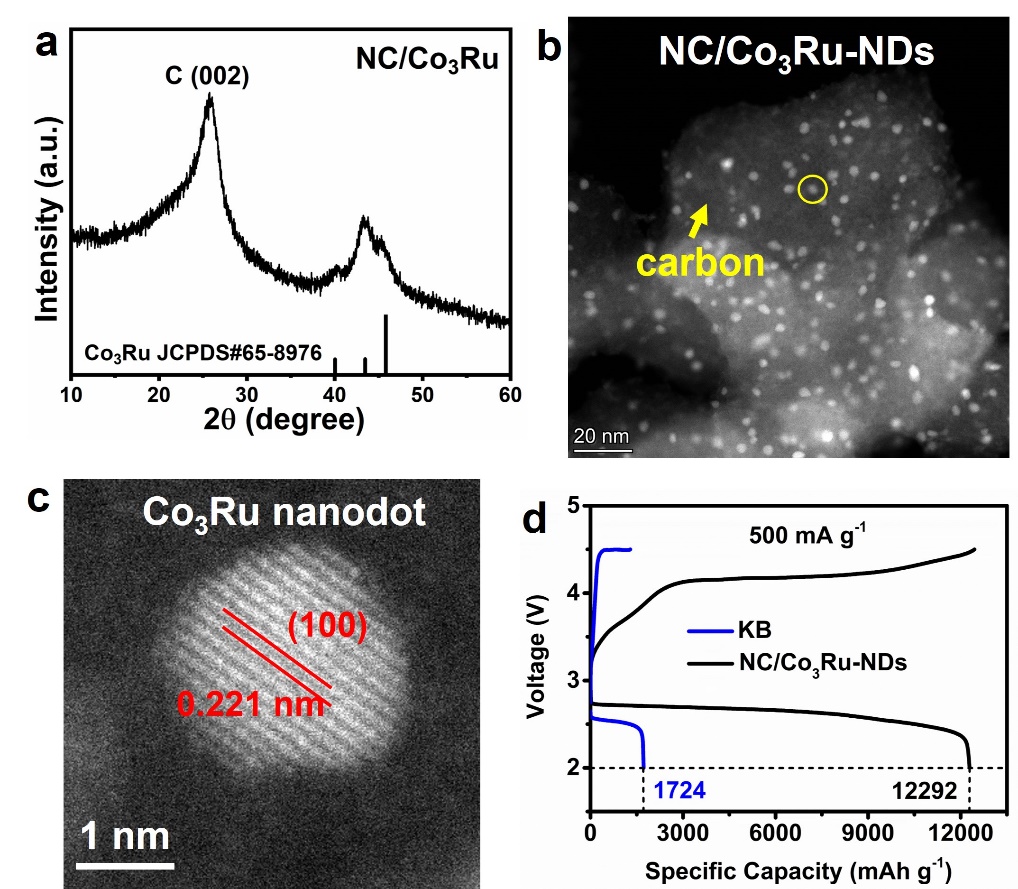


**Fig. S12** (**a**) XRD pattern, (**b**) HAADF-STEM image and (**c**) AC-STEM image of NC/Co_3_Ru-NDs. (**d**) The initial deep discharge-charge curves of KB- and NC/Co_3_Ru-NDs-based LOBs at the current density range of 500 mA g^-1^ under 0.1 MPa

The phase composition and microstructure of NC/Co_3_Ru-NDs have been measured. The XRD pattern shows that the catalyst is composed of carbon and Co_3_Ru intermetallic compound (JDPDS#65-8976). The high-angle annular dark-field scanning transmission electron microscope (HAADF-STEM) observation demonstrates that Co_3_Ru nanodots are dispersedly and uniformly embedded in a carbon matrix. They are 2-4 nm in size. The aberration-corrected STEM (AC-STEM) image of individual nanodot shows an interlayer spacing of 0.221 nm, corresponding to (100) plane of Co_3_Ru. The NC/Co_3_Ru-NDs catalyst cathode delivers remarkable improvement of discharge specific capacity in LOBs compared to KB-based LOBs.


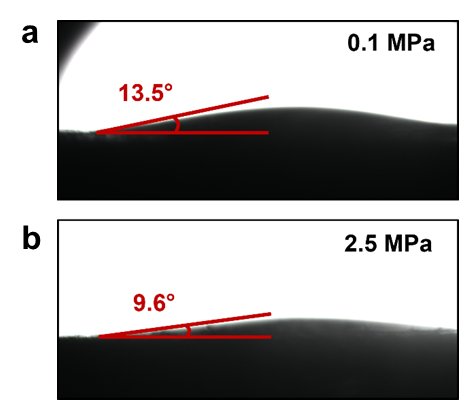


**Fig. S13** Contact angles between the electrolyte and the catalyst cathode under (**a**) 0.1 and (**b**) 2.5 MPa


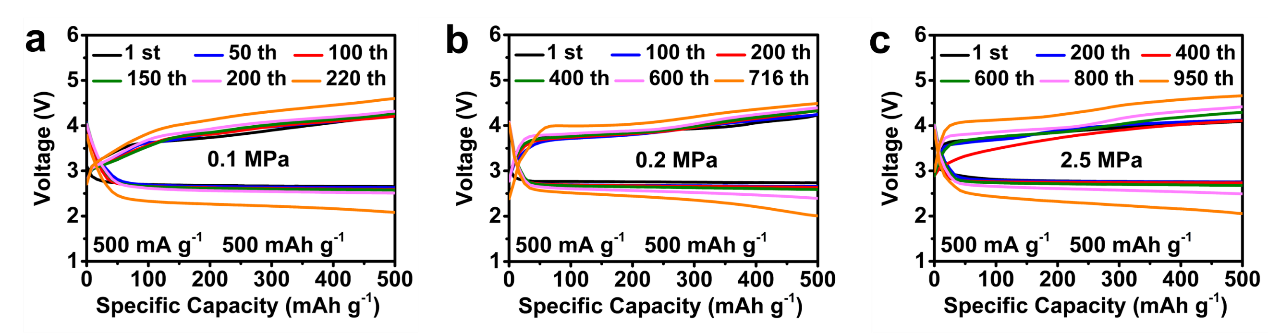


**Fig. S14** Cyclic stability of NC/Co_3_Ru-NDs-based LOBs at a current density of 500 mA g^-1^ with a limited capacity of 500 mAh g^-1^ under 0.1, 0.2 and 2.5 MPa


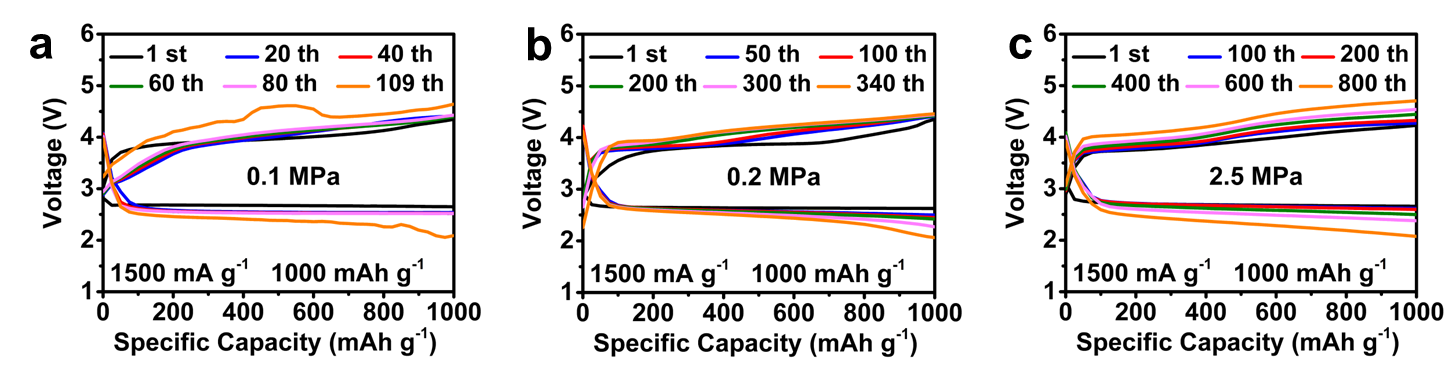


**Fig. S15** Cyclic stability of NC/Co_3_Ru-NDs-based LOBs with selected typical discharge/charge profiles at a current density of 1500 mA g^-1^ with a limited capacity of 1000 mAh g^-1^ under (**a**) 0.1, (**b**) 0.2 and (**c**) 2.5 MPa

**
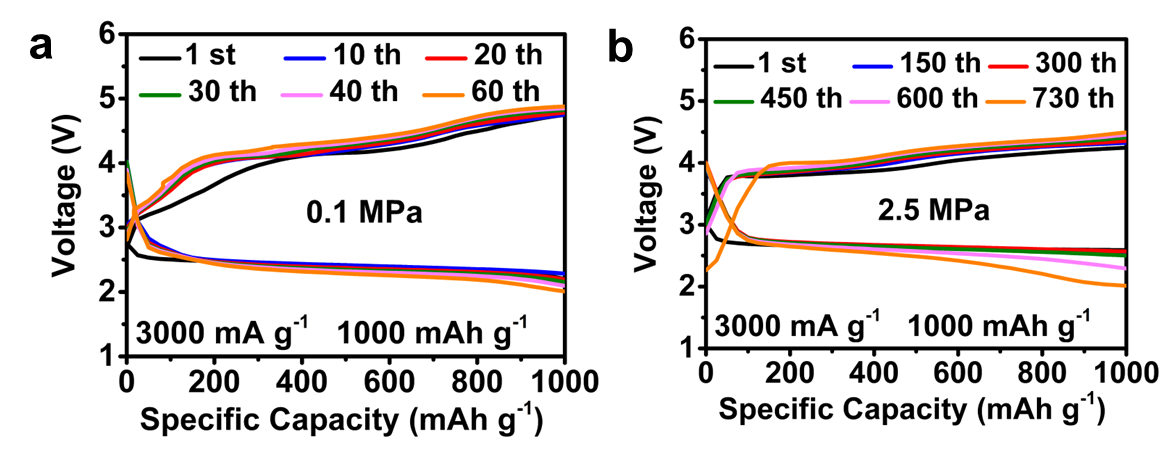
Fig. S16** Cyclic stability of NC/Co_3_Ru-NDs-based LOBs with selected typical discharge/charge profiles at a current density of 3000 mA g^-1^ with a limited capacity of 1000 mAh g^-1^ under (**a**) 0.1 and (**b**) 2.5 MPa


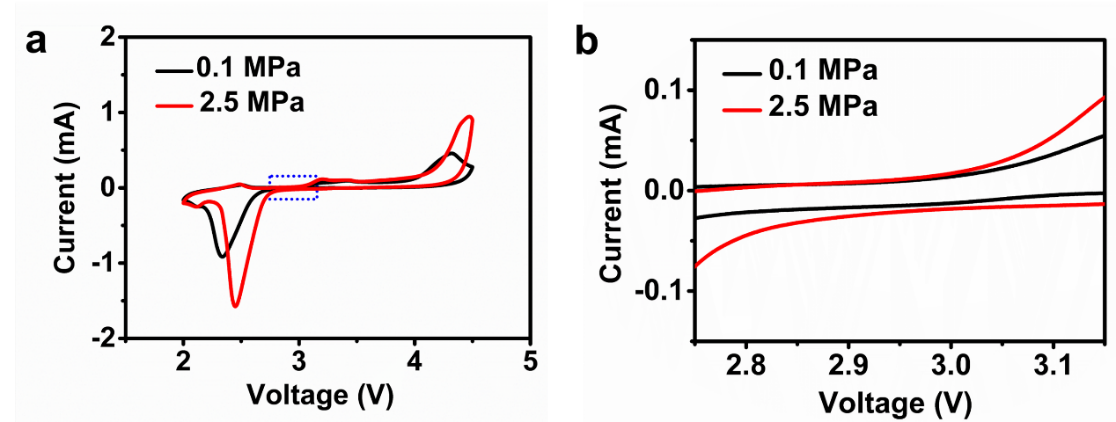


**Fig. S17** (**a**) CV curves of NC/Co_3_Ru-NDs-based LOBs at 0.5 mV s^-1^ under 0.1 (type B) and 2.5 MPa (type E). (**b**) The magnified view of the labeled voltage range


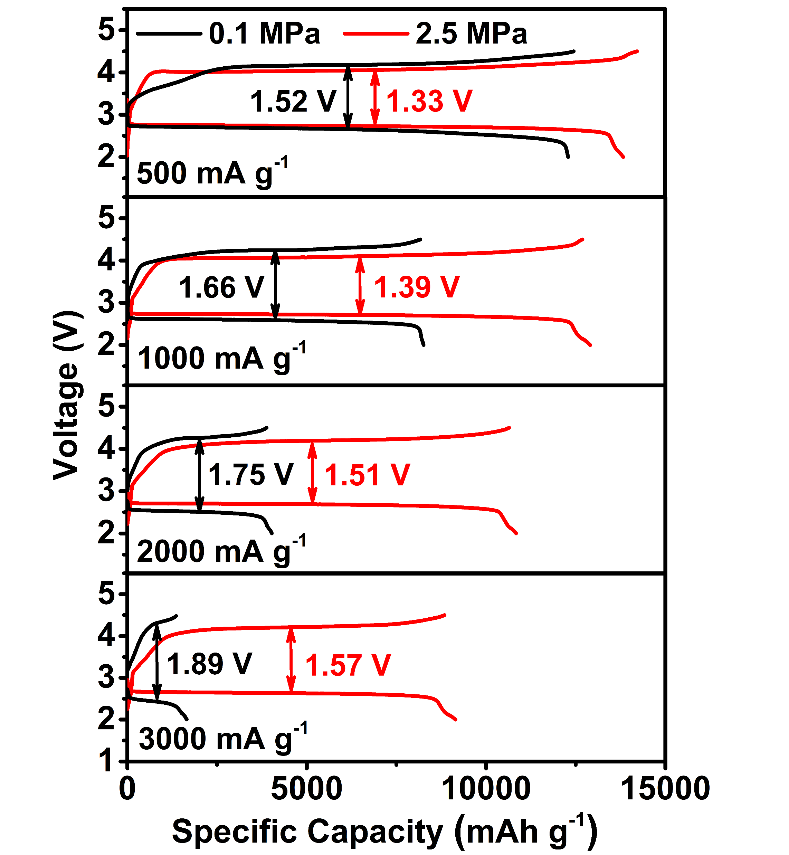


**Fig. S18** Initial deep discharge-charge curves and corresponding overpotentials of NC/Co_3_Ru-NDs-based LOBs at 500, 1000, 2000 and 3000 mA g^-1^ under 0.1 (type B) and 2.5 MPa (type D), respectively


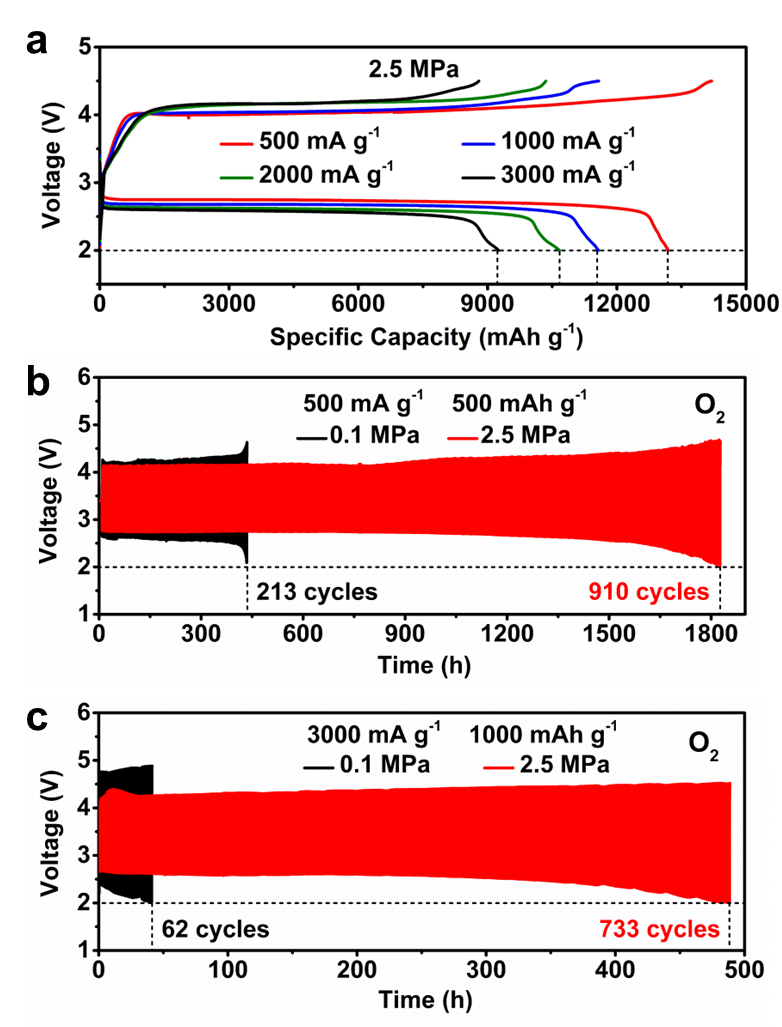


**Fig. S19** Repeatability of electrochemical properties of NC/Co_3_Ru-NDs-based LOBs with O_2_ compensation. (**a**) Capacity retention at different current densities under 2.5 MPa. Cycle stabilities at current densities of (**b**) 500 mA g^-1^ with a limited capacity of 500 mAh g^-1^ and (**c**) 3000 mA g^-1^ with a limited capacity of 1000 mAh g^-1^, respectively


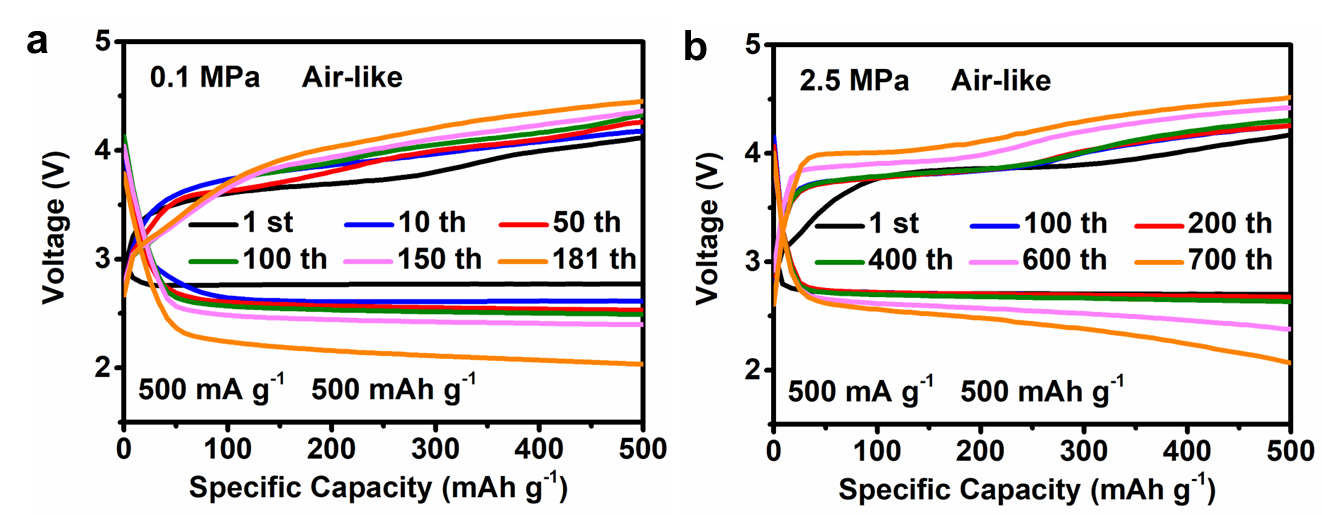


**Fig. S20** Cyclic stability of NC/Co_3_Ru-NDs-based LOBs with selected typical discharge/charge profiles at a current density of 500 mA ‍g^-‍1^ with a limited capacity of 500 mAh g^-1^ under (**a**) 0.1 and (**b**) 2.5 MPa in air-like atmosphere

**
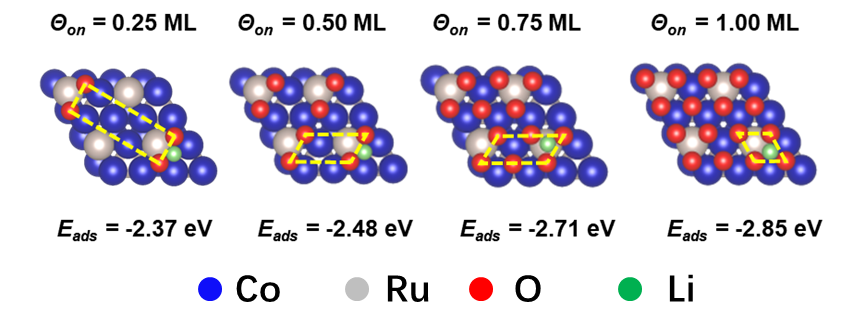
**

**Fig. S21** Schematic diagram of Li^+^ adsorption on the Co_3_Ru (002) surface with difference oxygen coverage of 0.25, 0.5, 0.75 and 1.0 ML, respectively

Because more dissolved oxygen is enriched on the surface of catalyst with O_2_-pressure elevation, the difference of O_2_ pressure is indirectly expressed by oxygen coverage (*Θ*_on_) difference in calculation. Four stable ordered surface phases with O (red ball) located on the (002) surface of Co_3_Ru catalysts, (4×1)-O, (2×1)-O, (4×1)-2O and (1×1)-‍O, correspond to 0.25, 0.50, 0.75 and 1.00 monolayer (ML) *Θ*_on_, respectively. The improved *Θ*_on_ positively gives rise to the increased adsorption energy (*E*_ads_) of Li^+^ (green ball) on the catalyst.


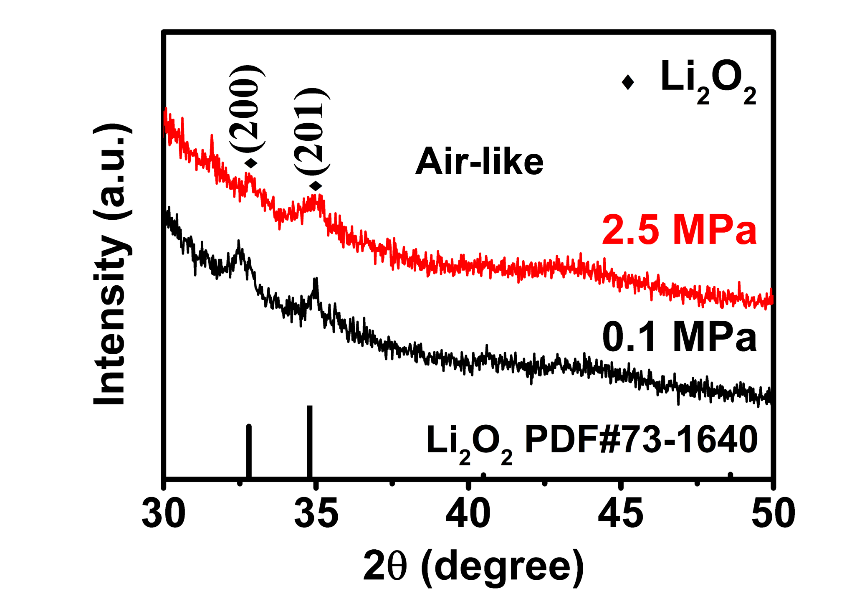


**Fig. S22** XRD patterns of discharged NC/Co_3_Ru-NDs cathodes under 0.1 and 2.5 MPa





**Fig. S23** EPR spectra of formed discharge products after discharged to 5000 mAh g^-1^ at 500 mA g^-1^ under 0.1 and 2.5 MPa


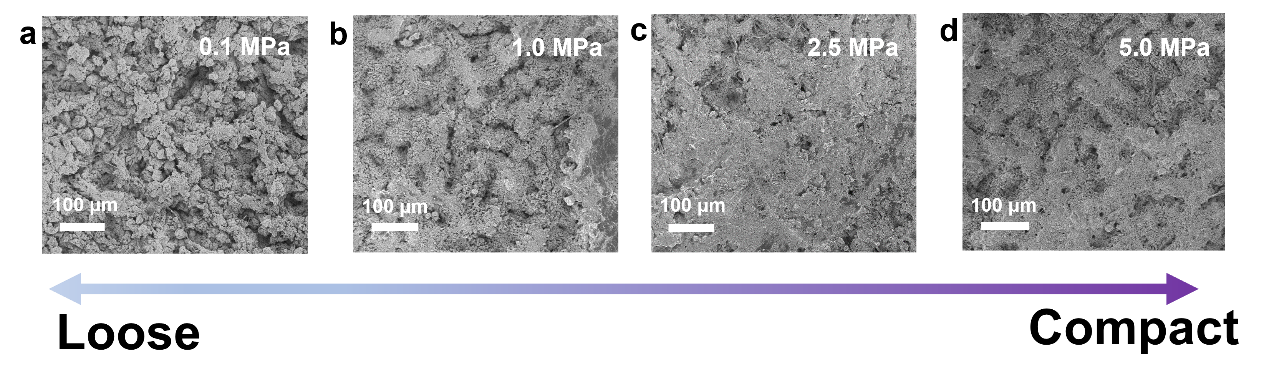


**Fig. S24** The top-view SEM images of the corrosion layers on anode surfaces after 15 cycles under (**a**) 0.1 MPa, (**b**) 1.0 MPa, (**c**) 2.5 MPa and (**d**) 5.0 MPa


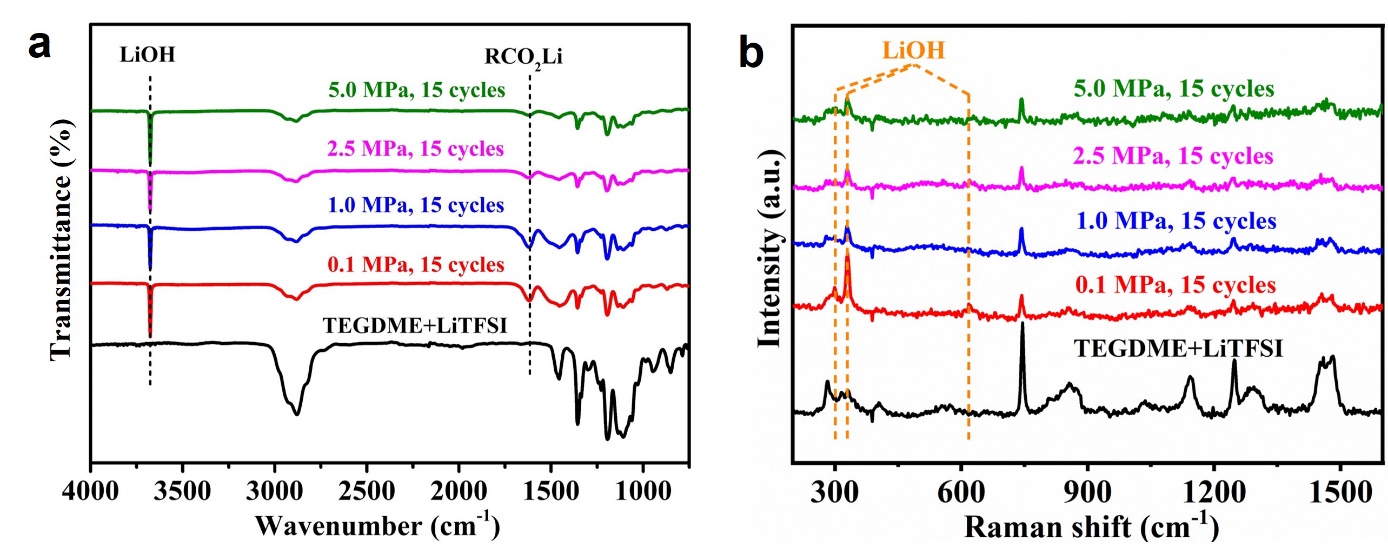


**Fig. S25** FTIR and Raman spectra of Li anodes obtained under different pressures

To investigate the positive effect of pressure on slowing down the corrosion rate of unprotected lithium anodes, the chemical compositions of the corrosion layer under different pressures are analyzed by FTIR and Raman spectra. After Li-O_2_ batteries are cycled 15 cycles at 500 mA g^-1^ with a cut-off capacity of 500 mAh g^-1^ under four different O_2_ pressures (0.1, 1.0, 2.5, and 5.0 MPa), these batteries are then disassembled and the anodes are characterized. As the FTIR and Raman spectra shown in Fig. S25, in addition to the peaks of the residual electrolyte (TEGDME+LiTFSI) on the anode surface, the signal of LiOH can be detected, indicating that the corrosion products on the anode are mainly composed of LiOH. In addition, the peak of LiOH in Raman spectra is smaller than 600 cm^-1^, corresponding to a long wavelength. This wavelength can penetrate a thin corrosion layer, so the Raman spectra are further utilized to preliminarily confirm the relative content of corrosion layers in anodes formed under different pressures. As shown in Fig. S25b, with the gradual increase of O_2_ pressure from 0.1 to 5.0 MPa, the Raman peak of LiOH significantly weakens, suggesting less corrosion products formed on the anode under higher pressure. It demonstrates that pressure has a role in delaying the corrosion rate of lithium anodes.

**
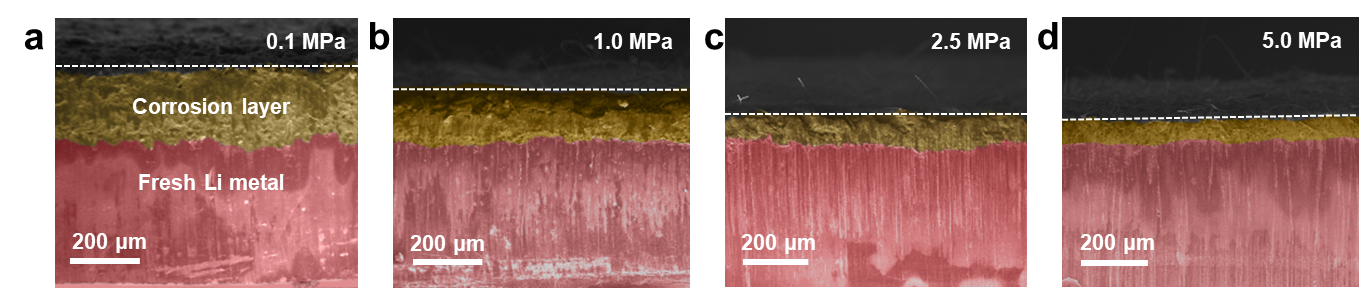
**

**Fig. S26** Cross-sectional SEM images of anodes after 15 cycles under (**a**) 0.1, (**b**) 1.0, (**c**) 2.5 and (**d**) 5.0 MPa

**
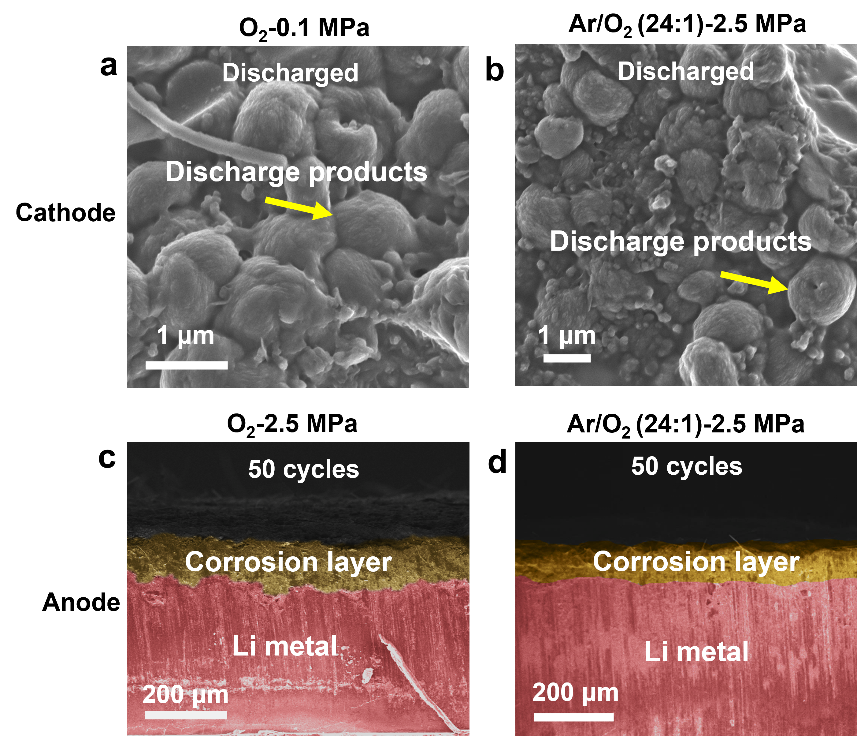
Fig. S27** SEM images of discharged cathodes (**a**) in 0.1 MPa O_2_, (**b**) in 2.5MPa Ar/O_2_ mixture (24:1). SEM images of anodes after 50 cycles (**c**) in 2.5 MPa O_2_, (**d**) in 2.5MPa Ar/O_2_ mixture (24:1)

A mixture of Ar and O_2_ with a ratio of 24:1 is used for battery tests instead of pure O_2_ under 2.5 MPa. Thus, the actual O_2_ partial pressure is 0.1 MPa in this case. The microstructure of formed discharge products is similar to that in type B batteries (0.1 MPa), further demonstrating the effect of actual O_2_ partial pressure in modulating the ORR kinetics. On the anode side, the feature of corrosion layers is analogical to that in type D battery (2.5 MPa).


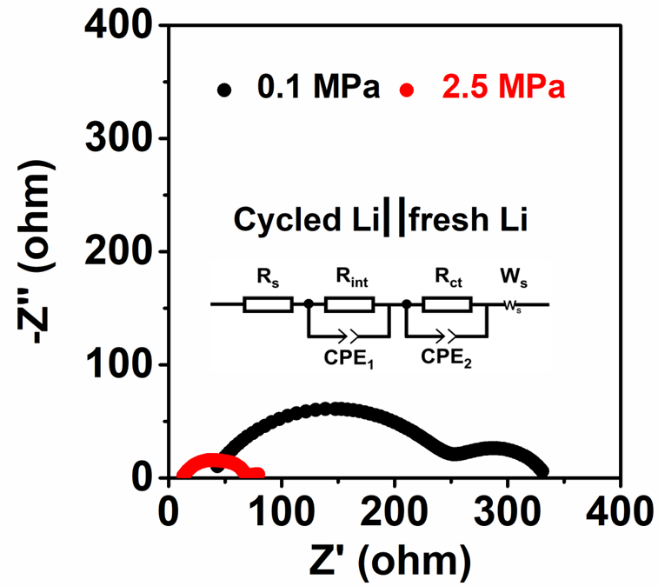


**Fig. S28** Electrochemical impedance spectroscopies of the Li||Li symmetric batteries under 0.1 (type B) and 2.5 (type D) MPa. The inset is the corresponding analog circuit diagram

The Li anode operated for 50 cycles in type B and D batteries, respectively, and a fresh Li wafer are reassembled into Li||Li symmetric batteries to investigate the charge transfer and Li^+^ diffusion kinetics of the corrosion layers. As shown in Fig. S28, the high- and medium-frequency capacitive arcs of EISs correspond to the migration process of Li^+^ within the corrosion layer and the charge transfer process of Li^+^ at the interface between the corrosion layer and fresh Li anode, respectively. It is clear that the EIS for the Li anode operated for 50 cycles under 2.5 MPa shows obviously decreased resistance compared to that under 0.1 MPa. The fitting results show that the *R_surface_* value of 49.64 Ω (type D) is much lower than that of 298.43 Ω (type B), indicating stronger migration of Li^+^ and charge-transfer kinetics of the Li anode in type D than in type B. This is attributed to remarkable thinning of corrosion layers for the former (Fig. 5e) *vs.* the later (Fig. 5h).


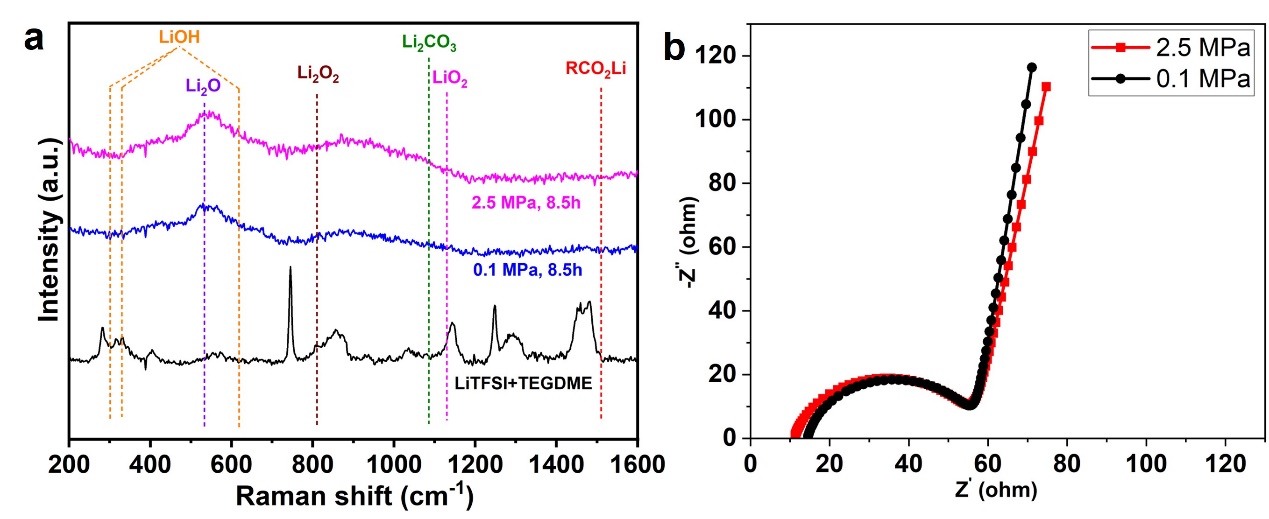


**Fig. S29** (**a**) Raman spectra and (**b**) electrochemical impedance spectroscopies of LOBs stood in O_2_ test chambers for 8.5 h under 0.1 and 2.5 MPa

High O_2_ pressure hardly affects the composition of SEI films. To exclude the influence from the protection layer, the effect for the formation of SEI films is investigated with bare Li anode. Generally, the assembled batteries are stably stood in O_2_ chambers for several hours before electrochemical test. A SEI film is formed on the surface of bare Li anode in this period. As shown in Fig. S29a, the SEI films are mainly composed of Li_2_O after 8.5 h under 0.1 and 2.5 MPa. They also present similar electrochemical impedance spectroscopies (EIS) and charge-transfer resistances (0.1 MPa: 41.7 Ω; 2.5 MPa: 44.8 Ω; Fig. S29b). Accordingly, the effect of SEI films for protecting Li anodes can be ignored during discharge/charge processes. In addition, when the batteries are operated 15 cycles under different pressures, only LiOH corrosion layer can be detected on Li metal anodes (Fig. S25). It demonstrates that the LiOH corrosion layer is rapidly formed instead of Li_2_O SEI film only after a couple of cycles. The protection for Li metal anode is limited via SEI films. It demonstrates the key role of the densification of corrosion layer for protecting bare Li anode but not SEI films.


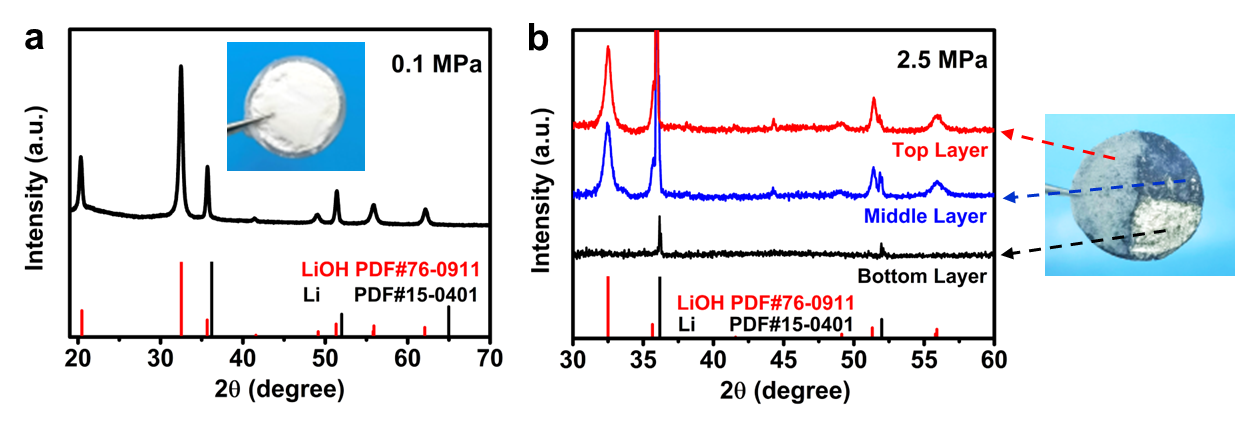


**Fig. S30** XRD patterns and optical photographs of anodes of LOBs after failure under (**a**) 0.1 MPa and (**b**) 2.5 MPa


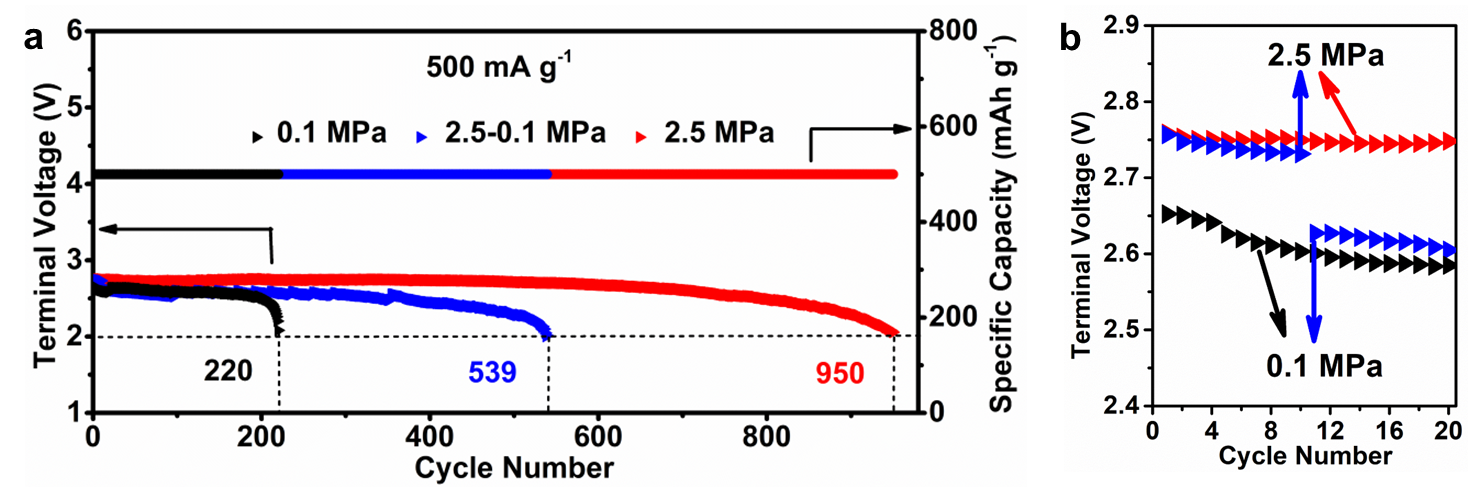


**Fig. S31** (**a**) Cycle stability of LOBs at 500 mA g^-1^ with a limited capacity of 500 mAh g^-1^ under 0.1, 2.5-0.1 and 2.5 MPa, respectively. (**b**) The initial 20 cycles in (a)


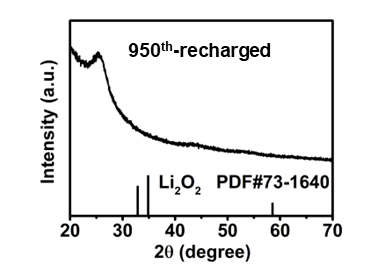


**Fig. S32** XRD pattern of the 950^th^ recharged cathode


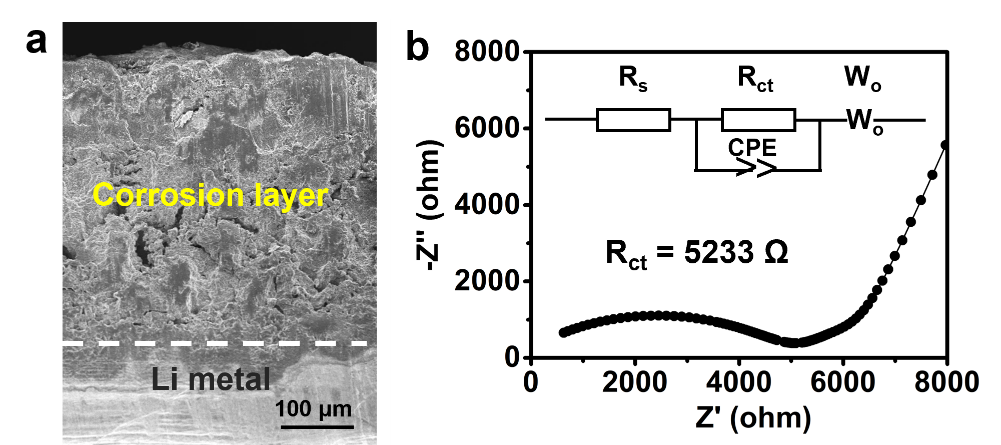


**Fig. S33** SEM image of the anode and the impedance diagram of the battery after failure under 2.5 MPa





**Fig. S34** Cycle performance of reassembled LOBs at 500 mA g^-1^ with a limited capacity of 500 mAh g^-1^ under 2.5 MPa


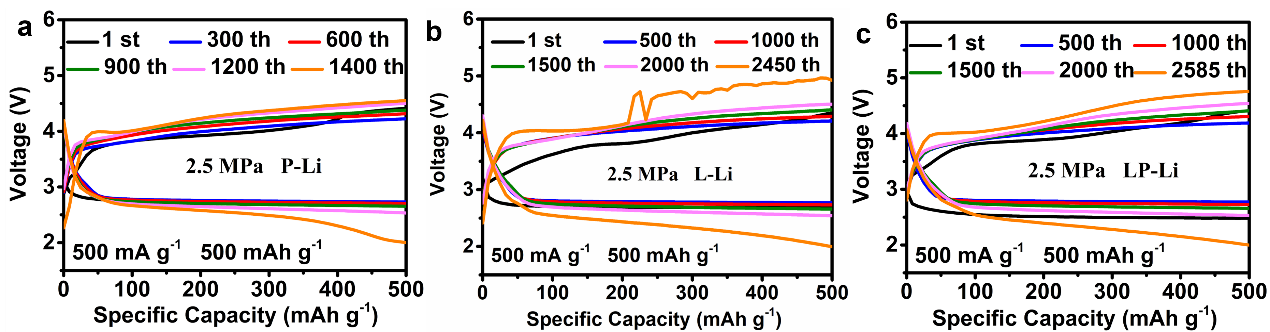


**Fig. S35** Cycling performance of P-Li, L-Li and LP-Li-based LOBs with selected typical discharge/charge profiles at a current density of 500 mA g^-1^ with a limited capacity of 500 mAh g^-1^ under 2.5 MPa


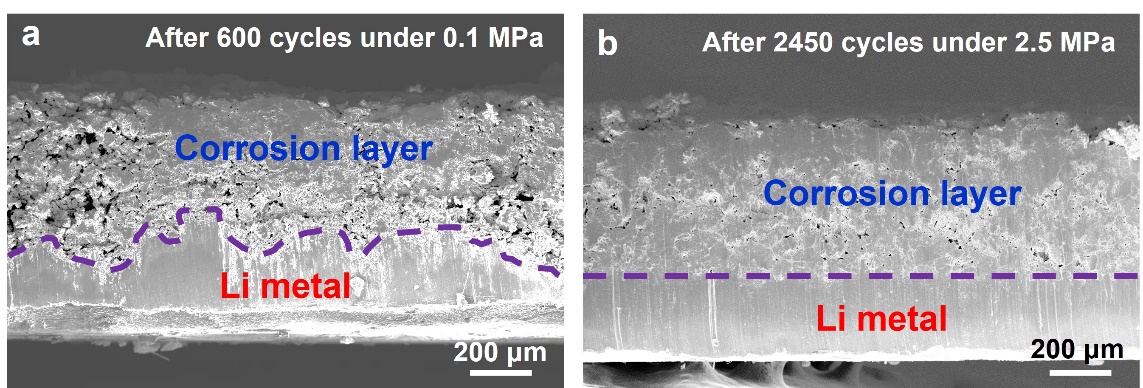


**Fig. S36** Cross-sectional SEM images of L-Li anodes under (**a**) 0.1 MPa after 600 cycles and (**b**) 2.5 MPa after 2450 cycles at 500 mA g^-1^ with a limited capacity of 500 mAh g^-1^





**Fig. S37** Cycle performance of NC/Co_3_Ru-NDs-based LOBs with LP-Li anode under 2.5 MPa at a current density of 500 mA g^-1^ with a limited capacity of 500 mAh g^-1^

**Table S1** Cycle performance comparison of this work with recently reported LOBs

| **Cathodes** | **Features** | | **Current**  **Density**  **(mA g^-1^)** | **Limited capacity**  **(mAh g^-1^)** | **Cycle**  **number** | **Refs.** |
| --- | --- | --- | --- | --- | --- | --- |
|  | | Redox mediator | 500 | 500 | 170 | [S6] |
| SnSe | |  | 500 | 600 | 380 | [S7] |
| Ru/rGO | | SEI film | 400 | 500 | 415 | [S8] |
| NiS_2_-CoS_2_@NC | |  | 500 | 500 | 490 | [S9] |
| Ni Foam@Co_3_O_4_-50RuO_2_ | | Gel polymer electrolyte | 500 | 500 | 553 | [S10] |
| CoNiO_2–x_F_x_/CC | |  | 500 | 500 | 580 | [S11] |
| MoO_2_-supported Mo_3_P@Mo | | Ni foam-gas diffusion layer | 500 | 500 | 685 | [S12] |
| MoS_2_ | | Lithium carbonate protection layer | 500 | 1000 | 700 | [S13] |
| Ni-RuO_2_ | |  | 500 | 500 | 791 | [S14] |
| Ru-MOF-C | |  | 500 | 800 | >800 | [S15] |
| Ag_2_Mo_2_O_7_ | |  | 500 | 500 | 816 | [S16] |
| Co_3_Ru-NC | | hybrid protection layer | 500 | 500 | 990 | [S17] |
| Mo_3_P | |  | 500 | 500 | 1200 | [S18] |
|  | | Overcharge + renewal anode + electrolyte addition | 500 | 500 | 1316 | [S19] |
| **Co_3_Ru-NC** | | **Pressure** | **500**  **1500** | **500**  **1000** | **950**  **800** | **This work** |
| **Co_3_Ru-NC** | | **Pressure + protection layer** | **500** | **500** | **2585**  **(5170 h)** | **This work** |

**Table S2** Comparison of *R*_ct_ at different discharge capacity of type B and D batteries

| **Battery** | **Discharge Capacity (mAh g^-1^)** | | | | |
| --- | --- | --- | --- | --- | --- |
|  | **1000** | **2000** | **3000** | **4000** | **5000** |
| Type B (0.1 MPa) | 40.34 Ω | 48.24 Ω | 55.56 Ω | 66.42 Ω | 75 Ω |
| Type D (2.5 MPa) | 39.8 Ω | 39.59 Ω | 40.84 Ω | 43.13 Ω | 47.12 Ω |

**Table S3** Comparison of *R*_int_, *R*_ct_ and *R*_surface_ of the cycled Li||fresh Li symmetric batteries under 0.1 and 2.5 MPa

| **O_2_ Pressure (MPa)** | ***R*_int_ (Ω)** | ***R*_ct_ (Ω)** | ***R*_surface_ (Ω)** |
| --- | --- | --- | --- |
| 0.1 | 214.5 | 83.93 | 298.43 |
| 2.5 | 40 | 9.64 | 49.64 |

**Supplementary References**

1. T. Lu, F. Chen, Multiwfn: a multifunctional wavefunction analyzer. J. Comput. Chem. **33**(5), 580–592 (2012). <https://doi.org/10.1002/jcc.22885>
2. L. Martínez, R. Andrade, E.G. Birgin, J.M. Martínez, PACKMOL: a package for building initial configurations for molecular dynamics simulations. J. Comput. Chem. **30**(13), 2157–2164 (2009). <https://doi.org/10.1002/jcc.21224>
3. G. Kresse, J. Furthmüller, Efficient iterative schemes for *ab initio* total-energy calculations using a plane-wave basis set. Phys. Rev. B **54**(16), 11169–11186 (1996). <https://doi.org/10.1103/physrevb.54.11169>
4. G. Kresse, D. Joubert, From ultrasoft pseudopotentials to the projector augmented-wave method. Phys. Rev. B **59**(3), 1758–1775 (1999). <https://doi.org/10.1103/physrevb.59.1758>
5. S. Grimme, J. Antony, S. Ehrlich, H. Krieg, A consistent and accurate *ab initio* parametrization of density functional dispersion correction (DFT-D) for the 94 elements H-Pu. J. Chem. Phys. **132**(15), 154104 (2010). <https://doi.org/10.1063/1.3382344>
6. Z. Sun, X. Lin, W. Dou, Y. Tan, A. Hu et al., Redox mediator with the function of intramolecularly disproportionating superoxide intermediate enabled high-performance Li–O2 batteries. Adv. Energy Mater. **12**(12), 2270050 (2022). <https://doi.org/10.1002/aenm.202270050>
7. G. Zhang, G. Li, J. Wang, H. Tong, J. Wang et al., 2D SnSe cathode catalyst featuring an efficient facet-dependent selective Li_2_O_2_ growth/decomposition for Li–oxygen batteries. Adv. Energy Mater. **12**(21), 2103910 (2022). <https://doi.org/10.1002/aenm.202103910>
8. X.-D. Lin, Y. Gu, X.-R. Shen, W.-W. Wang, Y.-H. Hong et al., An oxygen-blocking oriented multifunctional solid–electrolyte interphase as a protective layer for a lithium metal anode in lithium–oxygen batteries. Energy Environ. Sci. **14**(3), 1439–1448 (2021). <https://doi.org/10.1039/D0EE02931A>
9. D. Li, L. Zhao, J. Wang, C. Yang, Tailoring the d-band center over isomorphism pyrite catalyst for optimized intrinsic affinity to intermediates in lithium–oxygen batteriesrgy. Adv. Energy Mater. **13**(15), 2370060 (2023). <https://doi.org/10.1002/aenm.202370060>
10. C. Zhao, J. Liang, Q. Sun, J. Luo, Y. Liu et al., Ultralong-life quasi-solid-state Li-O_2_ batteries enabled by coupling advanced air electrode design with Li metal anode protection. Small Meth. **3**(2), 1800437 (2019). <https://doi.org/10.1002/smtd.201800437>
11. Z. Q. Sun, X. D. Lin, C. T. Wang, Y. Y. Tan, W. J. Dou et al., Constructing an interlaced catalytic surface via fluorine-doped bimetallic oxides for oxygen electrode processes in Li–O_2_ batteries. Adv. Mater., 2404319 (2024). <https://doi.org/10.1002/adma.202404319>
12. T. Yang, Y. Xia, T. Mao, Q. Ding, Z. Wang et al., Phosphorus vacancies and heterojunction interface as effective lithium-peroxide promoter for long-cycle life lithium–oxygen batteries. Adv. Funct. Mater. **32**(49), 2209876 (2022). <https://doi.org/10.1002/adfm.202209876>
13. M. Asadi, B. Sayahpour, P. Abbasi, A.T. Ngo, K. Karis et al., A lithium-oxygen battery with a long cycle life in an air-like atmosphere. Nature **555**(7697), 502–506 (2018). <https://doi.org/10.1038/nature25984>
14. C. Sun, X. Cui, F. Xiao, D. Cui, Q. Wang et al., Modulating the d-band center of RuO_2_ *via* Ni incorporation for efficient and durable Li–O_2_ batteries. Small **20**(32), 2400010 (2024). <https://doi.org/10.1002/smll.202400010>
15. X. Meng, K. Liao, J. Dai, X. Zou, S. She et al., Ultralong cycle life Li-O_2_ battery enabled by a MOF-derived ruthenium-carbon composite catalyst with a durable regenerative surface. ACS Appl. Mater. Interfaces **11**(22), 20091–20097 (2019). <https://doi.org/10.1021/acsami.9b05235>
16. H. Yu, G. L. Zhang, D. M. Zhang, R. N. Yang, X. Li et al., Homogeneous in-plane lattice strain enabling d-band center modulation and efficient d–π interaction for an Ag2Mo2O7 cathode catalyst with ultralong cycle life in Li-O_2_ batteries. Adv. Energy Mater. 2401509 (2024). <https://doi.org/10.1002/aenm.202401509>
17. F. Xiao, Q. Bao, C. Sun, Y. Li, D. Cui et al., D-band center regulation for durable catalysts and constructing a robust hybrid layer on Li anode enable long-life Li-O_2_ batteries. Adv. Energy Mater. **14**(15), 2303766 (2024). <https://doi.org/10.1002/aenm.202303766>
18. A. Kondori, Z. Jiang, M. Esmaeilirad, M. Tamadoni Saray, A. Kakekhani et al., Kinetically stable oxide overlayers on Mo_3_ P nanoparticles enabling lithium-air batteries with low overpotentials and long cycle life. Adv. Mater. **32**(50), e2004028 (2020). <https://doi.org/10.1002/adma.202004028>
19. K. Chen, D.-Y. Yang, J. Wang, G. Huang, X.-B. Zhang, Overcharge to remove cathode passivation layer for reviving failed Li–O_2_Batteries. CCS Chem. **5**(3), 641–653 (2023). <https://doi.org/10.31635/ccschem.022.202201876>
